# Comparison of life loss per death attributable to ambient temperature among various development regions: a nationwide study in 364 locations in China

**DOI:** 10.1186/s12940-020-00653-3

**Published:** 2020-09-15

**Authors:** Siqi Chen, Yize Xiao, Maigeng Zhou, Chunliang Zhou, Min Yu, Biao Huang, Yanjun Xu, Tao Liu, Jianxiong Hu, Xiaojun Xu, Lifeng Lin, Ruying Hu, Zhulin Hou, Junhua Li, Donghui Jin, Mingfang Qin, Qinglong Zhao, Weiwei Gong, Peng Yin, Yiqing Xu, Jianpeng Xiao, Weilin Zeng, Xing Li, Lingchuan Guo, Yonghui Zhang, Cunrui Huang, Wenjun Ma

**Affiliations:** 1 Guangdong Provincial Institute of Public Health, Guangdong Provincial Center for Disease Control and Prevention, No.160, Qunxian Road, Panyu District, Guangzhou, 511430 Guangdong China; 2 Yunnan Center for Disease Control and Prevention, Kunming, 650022 China; 3 The National Center for Chronic and Noncommunicable Disease Control and Prevention, Beijing, 100050 China; 4 Department of environment and health, Hunan Provincial Center for Disease Control and Prevention, Changsha, 450001 China; 5 Zhejiang Center for Disease Control and Prevention, Hangzhou, 310051 Zhejiang China; 6 Jilin Provincial Center for Disease Control and Prevention, Changchun, 130062 China; 7 Guangdong Provincial Center for Disease Control and Prevention, Guangzhou, 511430 China; 8 School of Public Health, Sun Yat-sen University, Guangzhou, 510080 China

**Keywords:** Temperature, Years of life lost, Mortality burden, Socioeconomic development level

## Abstract

**Background:**

Several studies have investigated the associations between ambient temperature and years of life lost (YLLs), but few focused on the difference of life loss attributable to temperature among different socioeconomic development levels.

**Objectives:**

We investigated the disparity in temperature-YLL rate relationships and life loss per death attributable to nonoptimal temperature in regions with various development levels.

**Methods:**

Three hundred sixty-four Chinese counties or districts were classified into 92 high-development regions (HDRs) and 272 low-development regions (LDRs) according to socioeconomic factors of each location using K-means clustering approach. We used distributed lag non-linear models (DLNM) and multivariate meta-analysis to estimate the temperature-YLL rate relationships. We calculated attributable fraction (AF) of YLL and temperature-related average life loss per death to compare mortality burden of temperature between HDRs and LDRs. Stratified analyses were conducted by region, age, sex and cause of death.

**Results:**

We found that non-optimal temperatures increased YLL rates in both HDRs and LDRs, but all subgroups in LDRs were more vulnerable. The disparity of cold effects between HDRs and LDRs was significant, while the difference in heat effect was insignificant. The overall AF of non-optimal temperature in LDRs [AF = 12.2, 95% empirical confidence interval (eCI):11.0–13.5%] was higher than that in HDRs (AF = 8.9, 95% eCI: 8.3–9.5%). Subgroups analyses found that most groups in LDRs had greater AFs than that in HDRs. The average life loss per death due to non-optimal temperature in LDRs (1.91 years, 95% eCI: 1.72–2.10) was also higher than that in HDRs (1.32 years, 95% eCI: 1.23–1.41). Most of AFs and life loss per death were caused by moderate cold in both HDRs and LDRs.

**Conclusions:**

Mortality burden caused by temperature was more significant in LDRs than that in HDRs, which means that more attention should be paid to vulnerable populations in LDRs in planning adaptive strategies.

## Introduction

Climate change is widely recognized as one of the major public health threats of the twenty-first century. The Intergovernmental Panel on Climate Change has projected that global warming is likely to reach 1.5° between 2030 and 2052 if climate change continue the current rate [1]. A link between ambient temperature and mortality has been reported in numerous studies [2, 3]. Those studies have found that temperature-mortality curves are usually U or J shaped, which means that non-optimal temperatures, both low and high, lead to excess mortality [2]. Other studies further showed that socioeconomic status such as individual income and education attainment had a significant modifying effect on the association [4, 5].

Most previous studies used death count as a primary health outcome to examine the effects of ambient temperature exposure [2, 6]. This indicator takes only the number of deaths into account and not the age at death. Therefore, it does not represent the real burden of exposure to ambient temperature. Years of life lost (YLLs), an important component of disability adjusted life years (DALY), is an indicator of premature death used to evaluate the burden of disease. YLL is a more informative indicator because it puts more weight on death at young age than death at old age [7]. However, very few studies estimated the effects of temperature on YLLs, and most of them focused on urban areas [8]. Moreover, in previous studies, YLLs caused by temperature could not be comparable because total YLLs largely depends on the population size of each study location. YLLs should be adjusted by population, such as using YLL rate (i.e., YLL/10^5^ population), for comparison among different regions and populations.

China is a vast country where socioeconomic status is geographically heterogeneous. Most studies have recognized that socioeconomic factors are very important health determinants. Several studies have reported that socioeconomic factors independently affected the association of temperature with mortality counts [5, 9, 10]. For instance, several studies reported that temperature-mortality relationships had difference between urban and rural areas [11–13], gross domestic product (GDP) and average educational years also could explain spatial heterogeneity of temperature-related effects [10, 14, 15]. Based on these, we hypothesize that low socioeconomic development regions (LDRs) maybe more vulnerable to ambient temperature than high development regions (HDRs), which has not been examined in the previous studies at national level.

To test the hypothesis, we conducted a national study including 364 locations in China. We first classified the locations into different development levels regions according to urbanization level, level of educational attainment and GDP per capita of each location using K-means clustering algorithm. Then, we compared temperature-YLL rate associations and life loss per death attributable to non-optimal temperature among different development levels. Our findings will be helpful to identify vulnerable populations in regions requiring protection.

## Material and methods

### Study locations

**Fig. 1:**
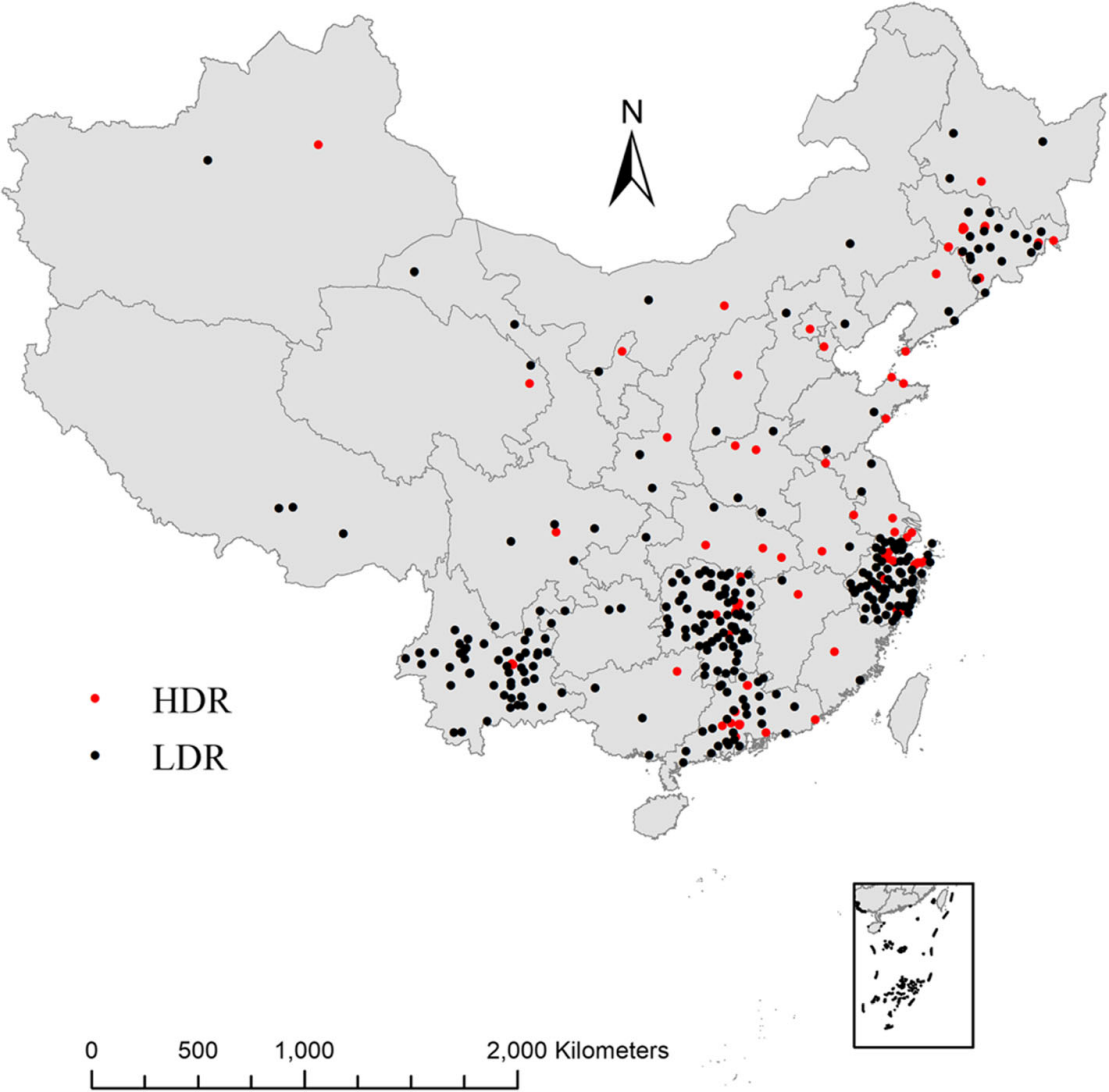
Distribution of 92 HDRs and 272 LDRs in the present study. HDR: high-development region; LDR: low-development region

The study included 364 counties or districts covering seven geographical regions around mainland China (Fig. 1). Study locations in Yunnan, Guangdong, Hunan, Zhejiang, and Jilin provinces were selected based on the provincial mortality surveillance system. Locations in other provinces were selected based on the China’s Disease Surveillance Points System (DSPs). DSPs is administrated by the Chinese Center for Disease Control and Prevention (CDC), and the detailed information on DSPs was described elsewhere [16, 17]. In order to assure enough statistical power, only locations with a population size over 200,000 or mortality rates larger than 4‰ were included [18]. The 364 locations were classified into 92 HDRs and 272 LDRs according to percentage of urban dwellers, average years of education and GDP per capita of each location using clustering methods. The detailed information on development level is described below.

Socioeconomic development is a comprehensive concept including social, economic, humanities development. Since many socioeconomic indicators at the county or district level is hard to obtain in China, we collected three available and representative socioeconomic indicators of each location, including percentage of urban dwellers for urbanization level, average years of education for education level and GDP per capita for economic level to evaluate socioeconomic development level. In order to ensure data-driven rather than arbitrary classification of development level, we employed K-means clustering analysis to classify development level. The K-means clustering algorithm is an unsupervised machine learning method for data clustering analysis. The main idea is the minimization of the distance within the clusters utilizing an iterative method [19]. In our study, the dataset was first divided into K clusters according to an arbitrarily chosen initial cluster center with the minimum distance principle; the average of each cluster then become new cluster centers for re-dividing. In such an iterative manner, the final clustering was developed when the cluster centers no longer changed. We determined the optimal number of clusters based on Nbclust, an R package which provides 30 indices for determining the best number of clusters and proposes the best clustering scheme from the different results obtained by varying all combinations of number of clusters (from 2 to 7) and distance measures [20]. The results showed that two clusters are optimal (Figure S1, see Supplementary material), which represents for HDRs and LDRs (Table S1, see Supplementary material).

### Data collection

Daily death records in locations from Yunnan, Guangdong, Hunan, Zhejiang, and Jilin provinces (from January 1, 2013 to December 31, 2017) were collected from the corresponding provincial CDC and daily death data of other locations (from January 1, 2006 to December 31, 2011) were collected from Chinese CDC, as described previously [18]. All deaths were classified based on the sole primary diagnosis coded by International Classification of Diseases, 10th Revision (ICD-10) including total non-accidental causes (codes: A00-R99), cardiovascular disease (CVD, codes I00-I99) and respiratory disease (RESP, codes J00-J98).

We calculated individual YLLs by matching the age and sex of every death to the life table for corresponding province (Table S2, see Supplementary material). The provincial life table was calculated based on the mortality data and demographical information collected from 2010 Population Census of China. Then daily YLLs of each location were quantified by summing all individual YLLs on the same day. We stratified daily YLLs by cause of death (non-accidental causes, CVD or RESP), sex (male or female), age (0–64 or 65+ years old), and region (northern China or southern China, divided by the north-south demarcation zone in China, which was developed based on the Geographic Information System with some quantitative methods) [21]. We further computed daily YLL rate (per 10^5^ populations) of each subgroup by dividing daily YLLs by the population size of the group, and multiplying 10^5^.

Daily mean temperature and relative humidity data from 698 climate stations across China were derived from the China Meteorological Data Sharing Service System (http://data.cma.cn/, Figure S2, see Supplementary material). We employed the Australian National University Splines (ANUSPLIN), an interpolation package based on the thin plate smoothing spline function, to interpolate the daily temperature grid and daily relative humidity grid at 0.01° × 0.01° resolution for all of China from 698 daily weather station observations. Longitude and latitude were considered as independent spline variables and elevation was regarded as a covariate. Ten-fold cross-validation confirmed the good prediction accuracy of the interpolation method for daily mean temperature [R^2^ = 0.96, root mean squared prediction error (RMSE) = 2.37 °C] and daily relative humidity (R^2^ = 0.81, RMSE = 7.7%) (Figure S3, see Supplementary material). Daily mean temperatures and relative humidity data from all 364 studied locations were extracted from the corresponding interpolated grid.

Since PM_10_ was the only available air pollutant during the whole study period (2006–2017), so we used it as a represent of air quality. Daily average PM_10_ data during 2006–2017 were obtained from the China National Environmental Monitoring Centre. Since the air quality monitoring system did not cover all study locations, we employed a random forest model to predict the daily PM_10_ of each location using the following predictors: daily mean temperature, daily RH, latitude, longitude, altitude, population density, length of road, types of land use and GDP per capital at each monitoring station using a radius of 1300 m [22, 23]. One smooth temporal basis function was also included in the model to control long-term and seasonal trend of PM_10_ concentrations. The result of ten-fold cross-validation for the model showed that the R^2^ was 0.78 and RMSE was 13.2 μg/m^3^(Figure S3, see Supplementary material). The population density data in 2015 were obtained from GeoData Institute in University of Southampton (www.worldpop.org.uk), and the geographic information system (GIS) (geographic map, road density, land use data and GDP per capita) were obtained from the Data Center for Resources and Environmental Sciences (http://www.resdc.cn).

GDP data and population data for each location were gathered from the Statistical Yearbook at the provincial and city level. Other county-level socioeconomic characteristics including average years of education and urban population size were obtained from the 2010 Population Census in China.

### Statistical analysis

#### Distributed lag non-linear model fitting

A two-stage analytic approach was employed to investigate the association between temperature and YLL rates. In the first stage, we estimated the location-specific association of temperature-YLL rate using distributed lag non-linear model framework (DLNM) [24] combined with Gaussian distribution function. The DLNM models were described as follows:

$$ E\left({Y}_t\right)=a+ cb\left({\mathrm{Tm}}_{\mathrm{t}},\mathrm{lag}\right)+ ns\left({\mathrm{t}\mathrm{ime}}_{\mathrm{t}}, df\right)+ ns\left({\mathrm{Rh}}_{\mathrm{t}}, df\right)+{\beta}_1{\mathrm{DOW}}_{\mathrm{t}} $$

where *Y*_t_ refers to the YLL rate on day t; *cb* (Tm_t_, lag) refers to the cross-basis function of daily temperature, defined as a quadratic B-spline with three knots (10th, 50th, 90th) of location-specific temperature distributions, and a natural cubic B-spline with an intercept and three internal knots placed at equally spaced values in the log scale; *ns* (time_t_, *df*) means natural cubic B spline function of time with 7 degrees of freedom (*df*) per year for the seasonal and long-term trend; *ns* (Rh_t_, *df*) means a natural cubic B-spline function of relative humidity with 3*df*; *β*_*1*_DOW_t_ refers to a categorical variable of day of the week. Previous studies suggested that effects of cold often appear several days later and last for about 2 weeks, and hence, we chose 21 days as the maximum lag [6].

As DLNM is a two-dimensional function considering non-linear exposure-response relationship and delayed effect simultaneously [24], we then reduced the full-association to the overall exposure-response association to cumulate full effects during the lag period. This step reduces the number of parameters for pooling in the second-stage meta-analysis.

In the second stage, we pooled the location-specific exposure-response associations using multivariate meta-analytical model with random effects models. The best linear unbiased prediction (BLUP) of location-specific cumulative associations between temperature and YLL rate were derived by use of the fitted meta-analytical model. The BLUP method makes use of a trade-off between the location-specific association and the pooled association to increase the preciseness of prediction, especially in location with small daily mortality counts [2].

#### Calculation of mortality burden of non-optimal temperatures

The minimum YLL rate temperature (MYT) and the corresponding minimum YLL rate temperature percentile (MYP) in each location were obtained from the corresponding BLUP of overall temperature-YLL rate associations and were defined as the optimal temperatures. We used the MYT as the reference for calculating the attributable YLL rate by re-centring the quadratic B spline that models the exposure-response. And the daily attributable YLL in each location was the production of attributable YLL rate and population size. The total attributable YLL was the sum of the attributable YLL for each day in time series. Its radio to the sum of YLLs during the study periods provides total attributable fraction (AF) [2], and its radio to the sum of daily death provides temperature-related life loss per death. We also estimated the AFs and life loss per death related to extreme cold, moderate cold, moderate heat, and extreme heat by summing the subsets of days with corresponding temperatures range (defined as≤2.5th percentile, 2.5th percentile to the MYT, MYT to 97.5th percentile, and ≥ 97.5th percentile of daily temperature, respectively). Empirical confidence intervals (eCIs) were obtained through Monte Carlo simulations with the assumption of a multivariate normal distribution of the BLUP of the reduced coefficients [25, 26].

### Sensitivity analysis

To check the robustness of all models, we conducted sensitivity analysis by varying the maximum lag periods and the *df* for time trend. We also investigate temperature-YLL rate using different temperature measures.

Since death datasets were collected from different data source, we perform a sensitivity analysis to compare the temperture-YLL rate associations in different death dataset.

R software version 3.6.0 was used to performed data analysis, with the “stats” package and “Nbclust” package for the K-means clustering algorithm, the “dlnm” package for constructing the DLNM model, and the “mvmeta” package for multivariate meta-analysis.

## Results

### Descriptive statistics

Summary statistics of daily YLL rates (per 10^5^), daily meteorological variables, and socioeconomic and demographic characteristics for HDRs and LDRs are presented in Table 1. The study had 10.7 million YLLs. Generally, there were higher average YLL rates in LDRs than in HDRs. Compared with HDRs, the daily mean temperature in LDRs was 1.0 degree Celsius higher. Socioeconomic factors in HDRs were notably higher than that in LDRs.

**Table 1:** Summary statistics of daily weather factors, YLL rate and socioeconomic characteristics

Development level	HDRs	LDRs
Number of locations	92	272
Daily YLL rate (Mean (SD))		
Total population	20.5(13.1)	24.7(16.6)
Age 0–64	12.0(11.8)	15.0(15.4)
Age65+	98.7(70.3)	110.9(75.5)
Males	24.3(19.2)	29.1(23.6)
Females	16.7(15.4)	20.1(19.9)
Cardiovascular	7.1(7.0)	8.7(8.5)
Respiratory	1.7(2.8)	2.6(4.4)
Northern China	21.0(14.4)	25.7(24.6)
Southern China	20.1(12.7)	24.5(14.8)
Daily mean temperature (°C)		
Mean (SD)	15.3(10.8)	16.3(9.5)
Minimum	−30.6	−32.3
2.5th percentile	−11.7	−6.2
25th percentile	8.3	10.1
50th percentile	17.1	17.7
75th percentile	23.7	23.4
97.5th percentile	30.6	30.2
Maximum	35.5	35.6
Relative humidity [%, Mean (SD)]	71.7(14.5)	73.6(13.7)
Socioeconomic and demographic characteristics		
Population (10^5^)	525.0	1501.5
Percent population of males [%, Mean (SD)]	50.5(2.3)	51.2(1.2)
Percent population >65y of age [%, Mean (SD)]	10.3(2.9)	10.2(2.5)
Percent of urban dweller [%, Mean (SD)]	86.3(14.1)	41.2(14.4)
Average years of education [Mean (SD)]	10.7(1.0)	8.3(0.8)
GDP per capita [thousand RMB, Mean (SD)]	55.2(34.1)	24.8(14.2)

Notes: *HDRs* High-development regions, *LDRs* Low-development regions, *SD* Standard deviation, *YLL rate* Years of life lost per 10^5^ populations, *GDP* Gross domestic product

### Temperature–YLL rate relationships

**Fig. 2:**
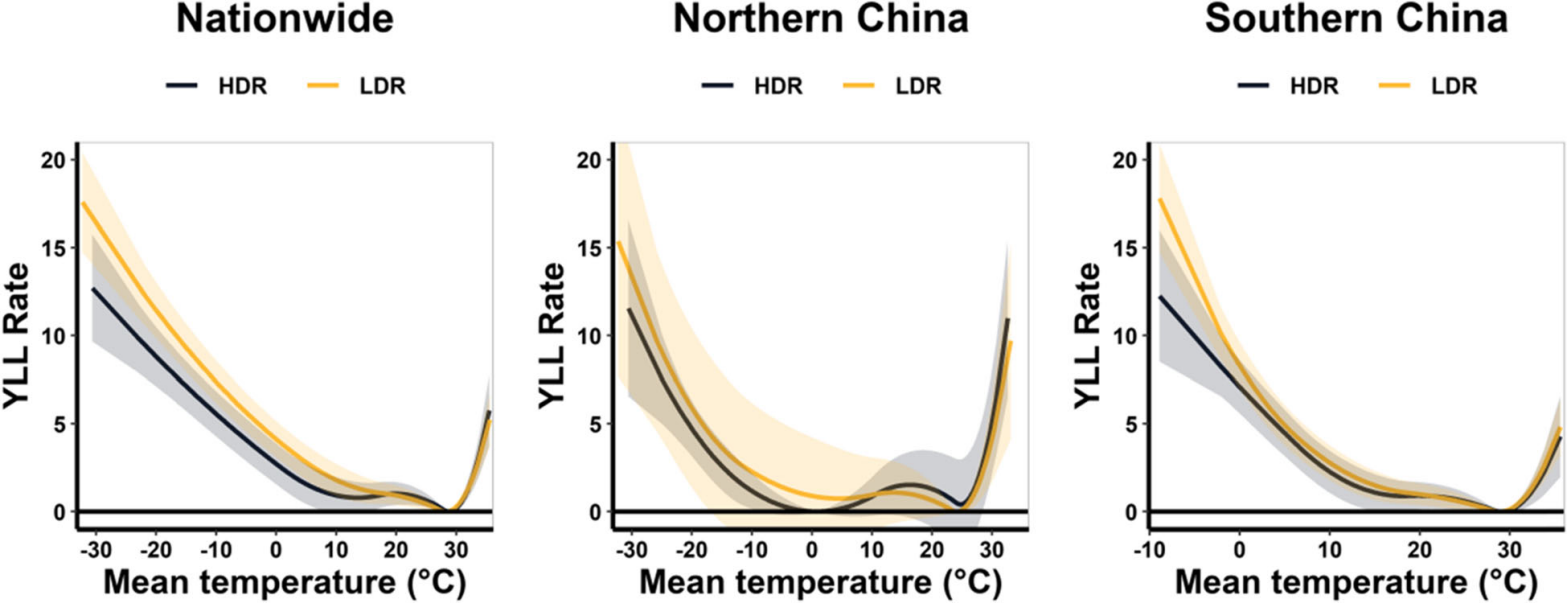
Overall cumulative temperature–YLL rate associations along lag 0–21 days with 95% confidence interval (CI). HDR: high-development region; LDR: low-development region; YLL rate: years of life lost per 10^5^ population

**Fig. 3:**
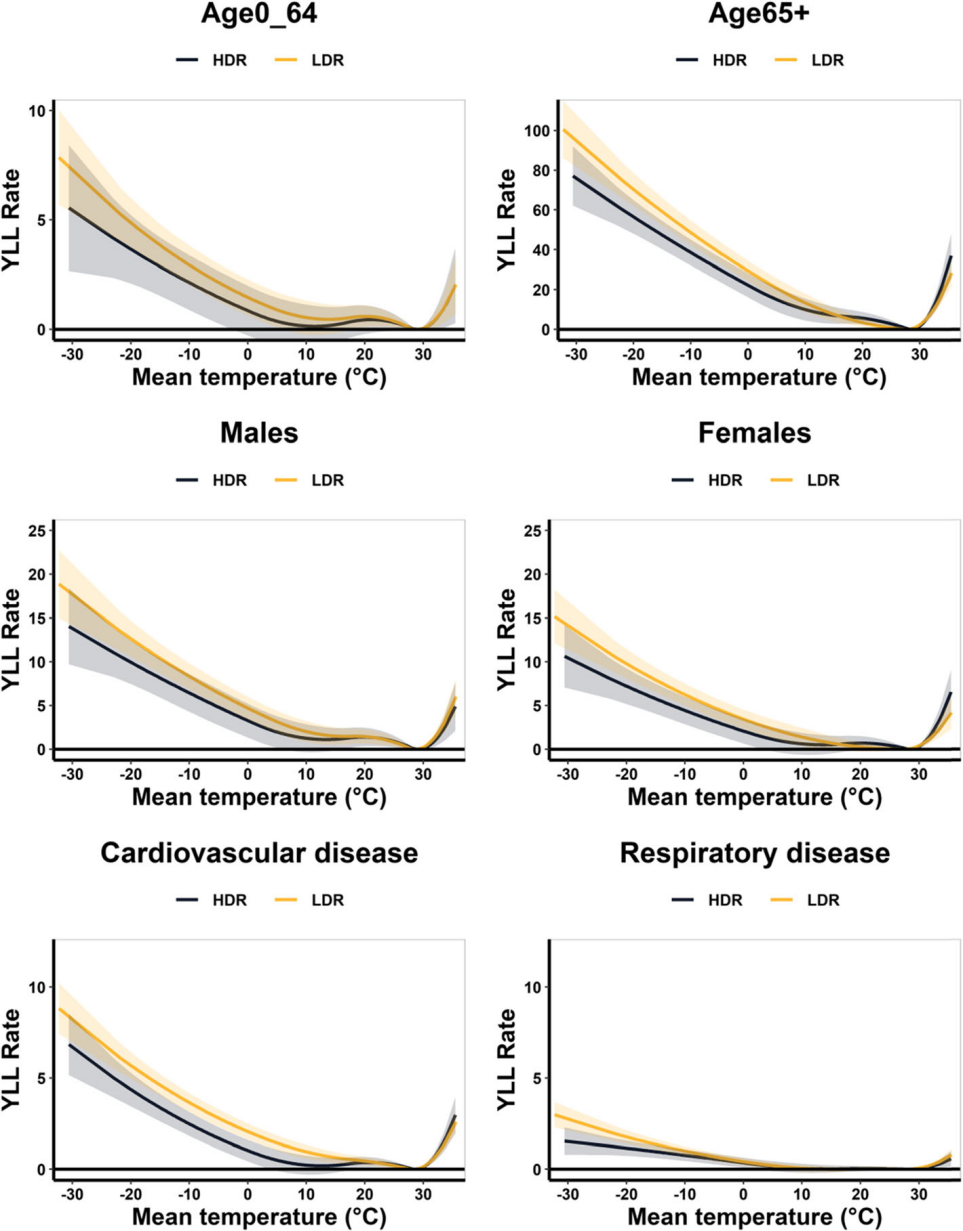
Pooled temperature–YLL rate associations along lag 0–21 days with 95% confidence intervals (CI) by age, sex, and cause of death in HDR and LDR. YLL rate: years of life lost per 10^5^ populations. HDR: high-development region; LDR: low-development region

Figure 2 shows the pooled associations between temperature and YLL rate for total population in LDRs and HDRs of China as a whole, northern China and southern China, and Fig. 3 displays the stratified analyses of the temperature-YLL rate associations in LDRs and HDRs by age, sex, and cause of death. For all above groups, we found that the attributed YLL rates below MYT were higher in LDRs compared with HDRs, while above MYT they were very close.

Table 2 further displayed the attributable YLL rates for extreme cold (2.5th percentile of temperature distribution) and extreme heat (97.5th percentile of temperature distribution). Compared with extreme heat-related YLL rate, the HDR-LDR disparity of extreme cold-related YLL rate were more significant. This HDRs-LDRs discrepancy for the elderly (≥65 years old) and cardiovascular disease was more distinct than that for young population and respiratory disease.

**Table 2:** Minimum YLL temperature (MYT) and attributable YLL (95%CI) rate associated with non-optimal temperatures by development level

Subgroups	MYT [°C, MYP (%)]		Extreme cold effect		Extreme heat effect	
	HDR	LDR	HDR	LDR	HDR	LDR
Nationwide	28.8(92.9)	28.3(92.5)	5.0(3.8–6.3)	6.7(5.5–7.9)	0.3(0.1–0.5)	0.4(0.2–0.6)
Northern China	0.8(28.8)	24.1(92.3)	3.8(2.4–5.3)	4.9(0.4–9.3)	1.0(−1.6–3.6)	0.7(0.2–1.3)
Southern China	29.1(93.5)	28.8(92.4)	5.8(4.4–7.2)	6.5(5.3–7.7)	0.2(0.0–0.5)	0.3(0.1–0.5)
Age 0–64	29.0(93.4)	28.9(94.3)	1.9(0.7–3.2)	2.6(1.8–3.5)	0.1(− 0.1–0.2)	0.1(0.0–0.2)
Age65+	28.5(92.0)	27.7(90.5)	35.5(29.2–41.9)	44.7(38.8–50.6)	2.4(1.3–3.4)	3.0(2.0–4.0)
Males	29.1(93.7)	28.7(93.7)	5.8(3.7–8.0)	7.6(6.1–9.0)	0.2(0.0–0.4)	0.3(0.1–0.5)
Females	28.5(92.0)	27.4(89.4)	4.0(2.4–5.5)	5.7(4.4–6.9)	0.4(0.2–0.7)	0.5(0.2–0.8)
Cardiovascular diseases	28.6(92.3)	28.3(92.5)	2.2(1.5–2.8)	3.3(2.8–3.8)	0.2(0.1–0.3)	0.2(0.1–0.3)
Respiratory diseases	28.4(91.7)	27.7(90.5)	0.7(0.4–1.0)	0.9(0.6–1.1)	0.0(0.0–0.1)	0.1(0.0–0.1)

Notes: *MYT* Minimum YLL rate temperature, *MYP* Minimum YLL rate percentile of the daily temperature

Extreme cold: 2.5th percentile of temperature distribution; nationwide: −8.2 °C, northern China: − 18.0 °C, southern China: 2.4 °C

Extreme heat: 97.5th percentile of temperature distribution; nationwide: 30.3 °C, northern China: 26.6 °C, southern China: 30.6 °C

### Comparison of temperature-related mortality burden between LDRs and HDRs

AFs and life loss per death associated with non-optimal temperatures in HDRs and LDRs are shown in Tables 3 and 4, respectively. Generally, temperature-related mortality burden was higher in LDRs compared with HDRs. The overall AFs were 8.9% (95% eCI: 8.3–9.5%) in HDRs and 12.2% (95% eCI: 11.0–13.5%) in LDRs, and temperature-related life loss per death were 1.32 years (95% eCI: 1.23–1.41) in HDRs and 1.91 years (95% eCI: 1.72–2.10) in LDRs. Cold-related AFs and life losses per death were higher in LDRs than those in HDRs for most populations excerpt patients with respiratory diseases. Cold-related AFs or life loss per death (below MYT) were much higher than heat-related AFs (above MYT) or life loss per death in both HDRs and LDRs.

**Table 3:** AFs attributable (95% eCI) to non-optimal temperatures by development level

Subgroups	Total		Cold		Heat	
	HDRs	LDRs	HDRs	LDRs	HDRs	LDRs
Nationwide	8.9(8.3–9.5)	12.2(11.0–13.5)	8.5(7.9–9.1)	10.4(9.3–11.4)	0.4(0.3–0.5)	1.9(1.1–2.6)
Northern China	7.3(4.8–9.7)	14.9(11.7–18.7)	4.0(2.3–5.7)	8.0(6.0–9.9)	3.3(1.4–5.1)	6.9(4.1–10.3)
Southern China	9.8(8.9–10.8)	12.5(11.1–13.9)	9.5(8.6–10.4)	10.7(9.4–11.8)	0.3(0.2–0.4)	1.8(1.0–2.6)
Age 0–64	6.5(3.7–9.1)	9.4(7.7–11.2)	5.3(3.7–6.7)	7.5(6.0–8.9)	1.3(− 1.0–3.4)	2.0(1.0–3.0)
Age65+	13.3(12.1–14.6)	16.2(15.0–17.4)	12.6(11.5–13.9)	14.4(13.4–15.4)	0.7(0.4–0.9)	1.8(1.0–2.6)
Males	10.2(8.7–11.7)	11.7(10.2–13.2)	9.6(8.2–11.0)	10.1(9.2–11.0)	0.6(0.2–1.0)	1.6(0.5–2.8)
Females	8.2(7.4–9.0)	11.1(9.6–12.6)	7.7(6.8–8.5)	9.6(8.3–10.9)	0.6(0.5–0.6)	1.5(0.7–2.5)
Cardiovascular diseases	11.3(9.7–12.9)	14.6(12.8–16.5)	9.8(8.2–11.2)	12.4(11.5–13.2)	1.6(0.9–2.2)	2.3(0.7–3.8)
Respiratory diseases	13.3(10.2–16.4)	13.9(10.1–17.3)	11.2(9.0–13.4)	8.0(6.9–9.0)	2.1(− 0.4–4.6)	5.9(2.4–9.2)

Notes: *HDRs* High-development regions, *LDRs* Low-development regions

Cold: temperature below minimum YLLs temperature; Heat: temperature above minimum YLLs temperature.

**Table 4:** Temperatures-related life loss per death (95% eCI) by development level

Subgroups	Total		Cold		Heat	
	HDRs	LDRs	HDRs	LDRs	HDRs	LDRs
Nationwide	1.32(1.23–1.41)	1.91(1.72–2.10)	1.26(1.17–1.35)	1.62(1.45–1.79)	0.06(0.05–0.08)	0.29(0.18–0.40)
Northern China	1.16(0.76–1.54)	2.58(2.03–3.23)	0.64(0.37–0.91)	1.38(1.04–1.72)	0.52(0.22–0.82)	1.38(1.04–1.72)
Southern China	1.42(1.29–1.56)	1.93(1.71–2.15)	1.38(1.24–1.51)	1.65(1.45–1.82)	0.04(0.03–0.06)	0.28(0.15–0.41)
Age 0–64	2.03(1.17–2.84)	3.03(2.48–3.58)	1.64(1.15–2.10)	2.40(1.94–2.87)	0.39(−0.32–1.05)	0.63(0.31–0.97)
Age65+	1.26(1.15–1.39)	1.59(1.47–1.71)	1.20(1.09–1.32)	1.41(1.31–1.52)	0.06(0.04–0.09)	0.18(0.1–0.10)
Males	1.57(1.33–1.79)	1.89(1.64–2.14)	1.48(1.26–1.69)	1.63(1.48–1.78)	0.09(0.03–0.15)	0.26(0.07–0.46)
Females	1.15(1.04–1.27)	1.66(1.42–1.87)	1.08(0.96–1.19)	1.43(1.23–1.61)	0.08(0.06–0.09)	0.22(0.10–0.37)
Cardiovascular diseases	1.42(1.21–1.61)	1.90(1.67–2.15)	1.22(1.02–1.40)	1.61(1.50–1.73)	0.20(0.11–0.28)	0.29(0.09–0.50)
Respiratory diseases	1.31(1.01–1.62)	1.53(1.11–1.90)	1.11(0.89–1.32)	0.88(0.76–0.99)	0.21(−0.03–0.45)	0.65(0.26–1.01)

Notes: *HDRs* High-development regions, *LDR*s Low-development regions

Cold: temperature below minimum YLLs temperature; Heat: temperature above minimum YLLs temperature.

**Fig. 4:**
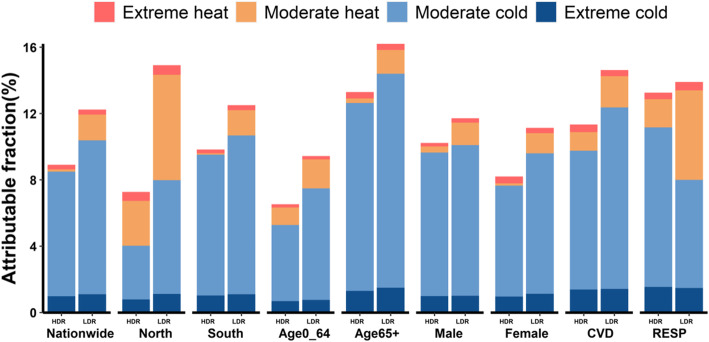
Attributable fraction of YLLs for separated temperature components. HDR: high-development region; LDR: low-development region; CVD: cardiovascular diseases; RESP: respiratory diseases

AFs and life loss per death were further estimated for four temperature components: extreme cold, moderate cold, moderate heat, and extreme heat (Fig. 4, Table S3, Table S4, see Supplementary material). Extreme temperature contributed to 0.19 years life loss per death in HDRs and 0.22 years life loss per death in LDRs. Moderate cold contributed to majority of mortality burden, with a total AF of 7.7% in HDRs and a total AF of 8.3% in LDRs, and an average of 1.11 years life loss per death from temperature in HDRs and 1.45 years life loss per death from temperature in LDRs were associated with moderate cold.

### Sensitivity analysis

Temperature-YLL rate curves for HDRs and LDRs were performed when varying the *df*s for time trend, lag structure and temperature measures (Figure S4, see Supplementary material). Figure S5 show temperature-YLL association for HDRs and LDRs in different datasets. The sensitivity analysis suggested that our main results were relatively stable.

## Discussion

To the best of our knowledge, this is the first multi-locations time-series study to investigate the disparity in temperature-YLL rate relationship and life loss per death attributable to temperature among different development regions in a developing country. We found an apparent disparity in YLL rates caused by temperature between HDRs and LDRs. Moreover, attributable YLL rates for cold temperature were higher in LDRs than that in HDRs, but heat-related YLL rates were very close between the two development regions.

Human Development Index (HDI) is a summary measure of human development in the literature [27]. In the study, we did not define development level using HDI for data unavailability, which may influence the comparison of our findings with other studies. However, the three indicators we chose to classify development levels are appropriate because they reflect different aspects of socioeconomic development level including economic status, urbanization level and education level. We classified all study locations into different development levels using K-means clustering algorithm rather than simple equisection of the three indicators because K-means is a scientific cluster analysis with a clear mechanism [28–30].

Our findings showed that the attributable YLL rates were greater in LDRs than that in HDRs for all subgroups. This finding is consistent with several previous studies that investigated urban-rural mortality disparity using relative risk (RR) [11–13]. For example, a study in Zhejiang, China found that the RRs associated with temperature were higher in rural areas than that in urban areas for all causes of death, the elderly (≥65 years old), and both sexes [12]. Hajat et al. found that lower economic status [e.g., gross domestic product (GDP)] associated with higher heat risk [14, 15]. Low average education also have been found worsen cold- and heat-related mortality burden [10]. Possible reasons for this finding were the potential differences in social environment and living conditions among different development regions such as population structure and density, lifestyle and availability to adequate health care services or home air conditioners. This result suggests that LDRs are more vulnerable to non-optimal temperature exposure and more resources should be allocated in these regions to protect vulnerable populations. For instance, it is necessary to improve health care services and provide adaptation assistance for vulnerable populations such as the elderly and patients with cardiopulmonary diseases in LDRs.

We further found that cold-related YLL rates were higher in LDRs compared with HDRs, which was consistent with previous findings that socioeconomic factors have a modifying effect on the temperature-mortality count relationship [10, 31]. However, the heat-related YLL rates were similar between HDRs and LDRs, which implies that development level does not play an important role in heat-related YLL rates. This result may partially be explained by urban heat-island (UHI) effect, which has been found to have an important impact on heat-related mortality [32]. Several previous studies reported that HDRs characterized by more urbanized areas and higher population densities were more vulnerable to heat [5, 10]. Though populations in HDRs might not be vulnerable to high temperature because of better adaption capacity [11, 33], the presence of UHI effect in HDRs may offset that capacity. In the context of rapid urbanization in most parts of China, effective public health policies designed to reduce UHI effects are necessary in HDRs to mitigate the health impacts of high temperature exposure.

We found that less YLLs attributed to non-optimal temperature in HDRs (8.8%) than in LDRs (10.4%). This is understandable because there are higher attributable YLL rates at each temperature in the LDRs compared with HDRs. We further calculated life loss per death attributed to non-optimal temperature and found they were high in HDRs than that in LDRs. Temperature-related life loss per death is a more informative indicator to understand health impact of temperature. Generally, we found that AF and life loss per death resulting from non-optimal temperature were greater in LDRs than that in HDRs in most subgroups. However, we found the opposite results for patients with RESP disease. This may be owing to AFs determined by both attributable YLL rates and temperature distribution. Compared with HDR, the attributable YLL rates of RESP in LDR are just little higher and were offset by a larger proportion of days around MYT with very low attributed YLL rates in LDRs (Figure S5, see Supplementary material), which might lead to lower attributable YLLs in LDRs for the RESP subgroups compared with HDRs.

We further calculated attributable YLLs and life loss per death for separated temperature components and the results showed that moderate cold was responsible for most attributable YLLs and life loss per death in both LDRs and HDRs. This finding is in line with previous studies based on death counts [2, 6]. However, most previous research focused on the health impact of extreme temperature and public health policies of adaptive interventions were designed mainly for extreme climate events [34]. In the future, more public health measures should be taken targeting moderate cold to effectively reduce temperature-related mortality burdens.

The present study has several strengths. First, a national multi-location analysis enabled us to synthetically estimate the effect of ambient temperature in various development level regions. Second, we utilized a population adjusted YLL rate to estimate the temperature-YLL associations, which allowed us to directly compare the effects of temperature exposure on YLLs among different regions. Last, we used the high-resolution interpolated temperatures and instead of fixed-site monitor data for temperature exposure of each location, which may reduce exposure measurement error.

Some limitations of our study cannot be ignored. First, it was an ecological study, and hence, some inherent limitations made us unable to take individual exposure into account. Second, there were not enough study locations in northwest China and southwest China because of the unavailability of mortality data. Third, the study periods in all locations were inconsistent due to the unavailability of death data. However, a study conducted in Shanghai indicated that both heat and cold effects on mortality did not substantially change during 2001–2012 [35].

## Conclusion

This study investigated the associations of temperature and YLL rate in different development regions. Compared with HDRs, for all subpopulations, cold-related YLL rates were higher in LDRs, while YLL rates attributable to heat were similar. Life loss per death attributable to non-optimal temperatures were more in LDRs than in HDRs, and most of them were from moderate cold. Our findings have important implications for developing targeted adaptive policies to reduce the temperature-related mortality burden in different development regions.

## Supplementary information

**Additional file 1.** Supplementary Material.

## Data Availability

Meteorological data can be accessed from the China Meteorological Data Sharing Service System (http://data.cma.cn/). The mortality data and R code of this study are available from the corresponding author (mawj@gdiph.org.cn), upon reasonable request.

## Abbreviations

AF: Attributable fraction
CDC: Center for Disease Control and Prevention
CVD: Cardiovascular disease
DALY: Disability adjusted life years
DLNM: Distributed lag non-linear models
DSPs: Disease Surveillance Points System
eCIs: Empirical confidence intervals
GDP: Gross domestic product
HDRs: High development regions
LDRs: Low socioeconomic development regions
MYT: Minimum YLL rate temperature
MYP: Minimum YLL rate temperature percentile
RESP: Respiratory disease
RMSE: Root mean squared prediction error
YLL: Years of life lost
