# Supplementary material for: Comparison of life loss per death attributable to ambient temperature among various development regions: a nationwide study in 364 locations in China
Source: Environ Health. 2020 Sep 15;19:98. doi: 10.1186/s12940-020-00653-3 (PMC7491140; doi:10.1186/s12940-020-00653-3)
Supplement: Supplementary file 1 — Additional file 1. Supplementary Material. [file 12940_2020_653_MOESM1_ESM.docx]

**Supplementary Material**

**Comparison of life loss per death attributable to ambient temperature among various development regions: a nationwide study in 364 locations in China**

**Content**

[1. Supplementary figures 1](#_Toc13658)

[Figure S1 Number of cluster indices for different numbers of clusters k 1](#_Toc737)

[Figure S2 Location of 698 climate stations across China 2](#_Toc25943)

[Figure S3 Ten-fold cross validation of interpolated daily temperatures and relative humidity predicted daily PM](#_Toc26633)_[10](#_Toc26633)_ [3](#_Toc26633)

[Figure S4 Pooled temperature–YLL rate associations using alternative model choice in the sensitivity analyses in HDR and LDR](#_Toc37) [4](#_Toc37)

[Figure S5 Daily mean temperature distributions in China 5](#_Toc15339)

[2. Supplementary table 6](#_Toc3637)

[Table S1 High development location and low development location 6](#_Toc4273)

[Table S2 Life table for provinces in China by age and sex 11](#_Toc16033)

[Table S3 Attributable fraction of YLL for separated temperature components in HDRs and LDRs. 15](#_Toc28760)

[Table S4 Life loss per death for separated temperature components in HDRs and LDRs. 16](#_Toc21904)

# Supplementary figures


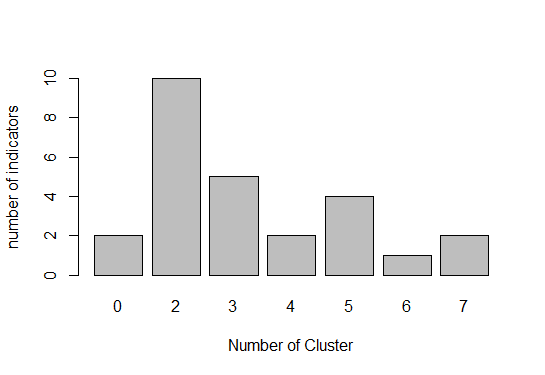


## Figure S1 Number of cluster indices for different numbers of clusters k


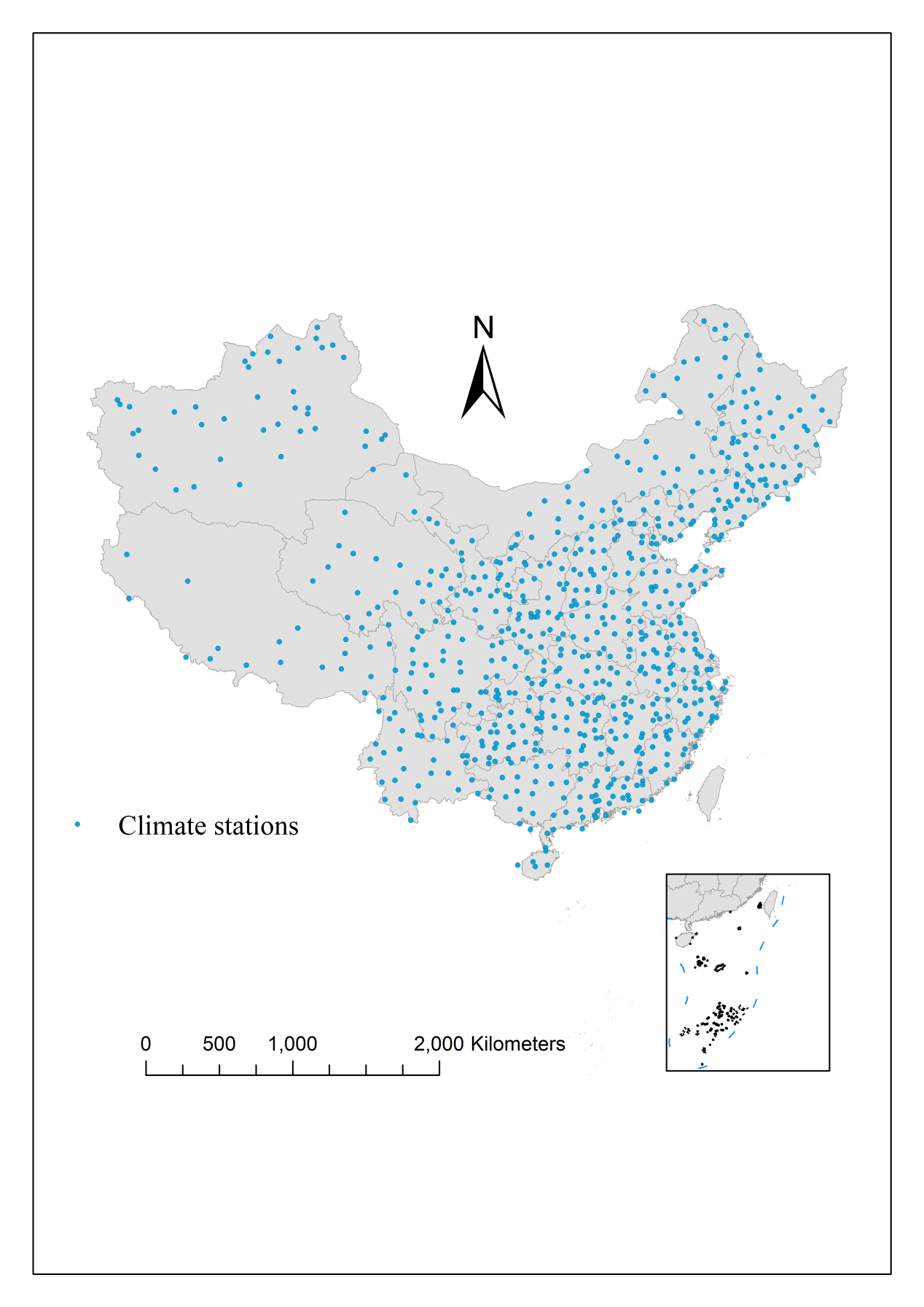


## Figure S2 Location of 698 climate stations across China


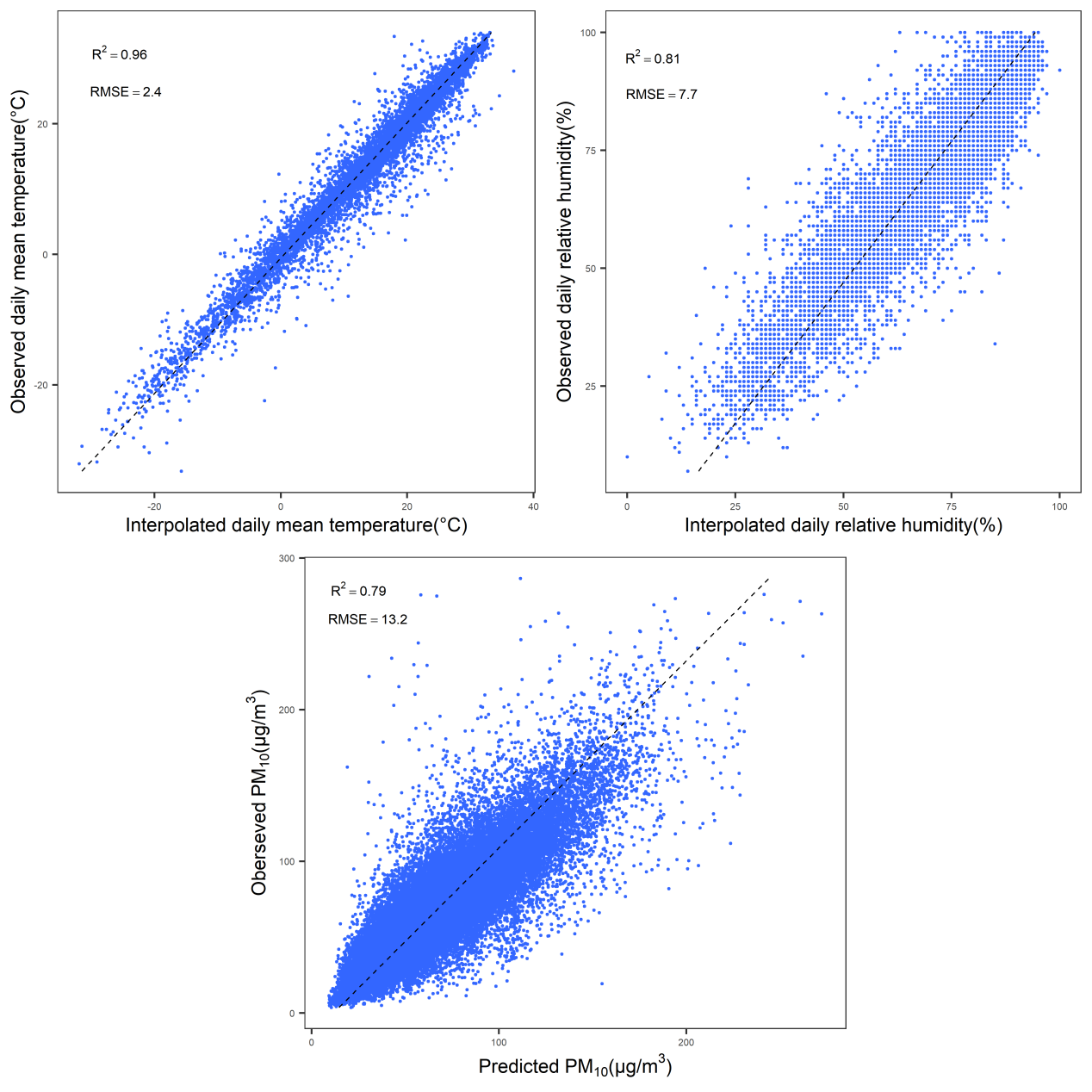


## Figure S3 Ten-fold cross validation of interpolated daily temperatures and relative humidity predicted daily PM_10_

**
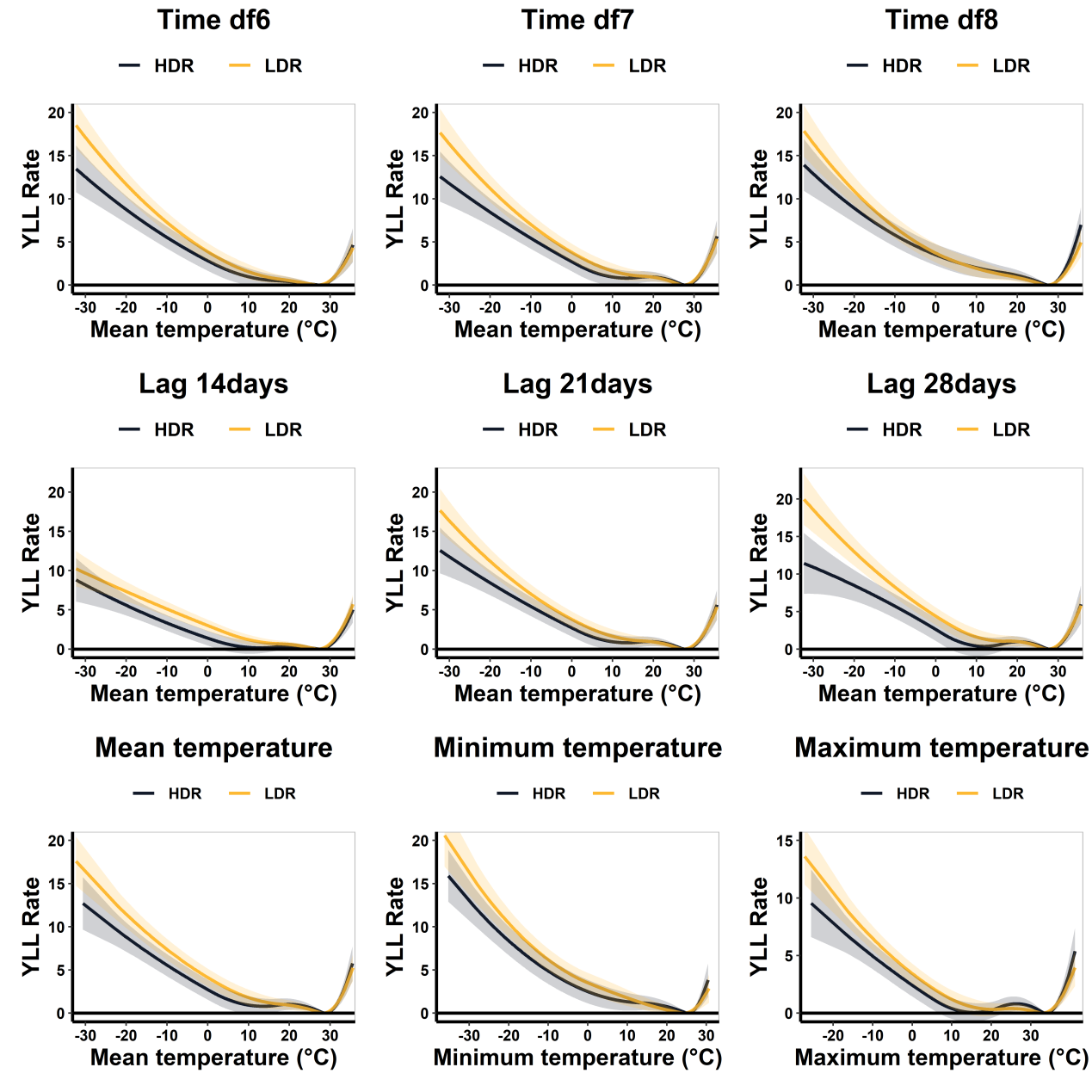
**

## Figure S4 Pooled temperature–YLL rate associations using alternative model choice and temperature measures in the sensitivity analyses in HDR and LDR

YLL rate: years of life lost per 10^5^ population; HDR: high-development region; LDR: low-development region; *df*: degrees of freedom


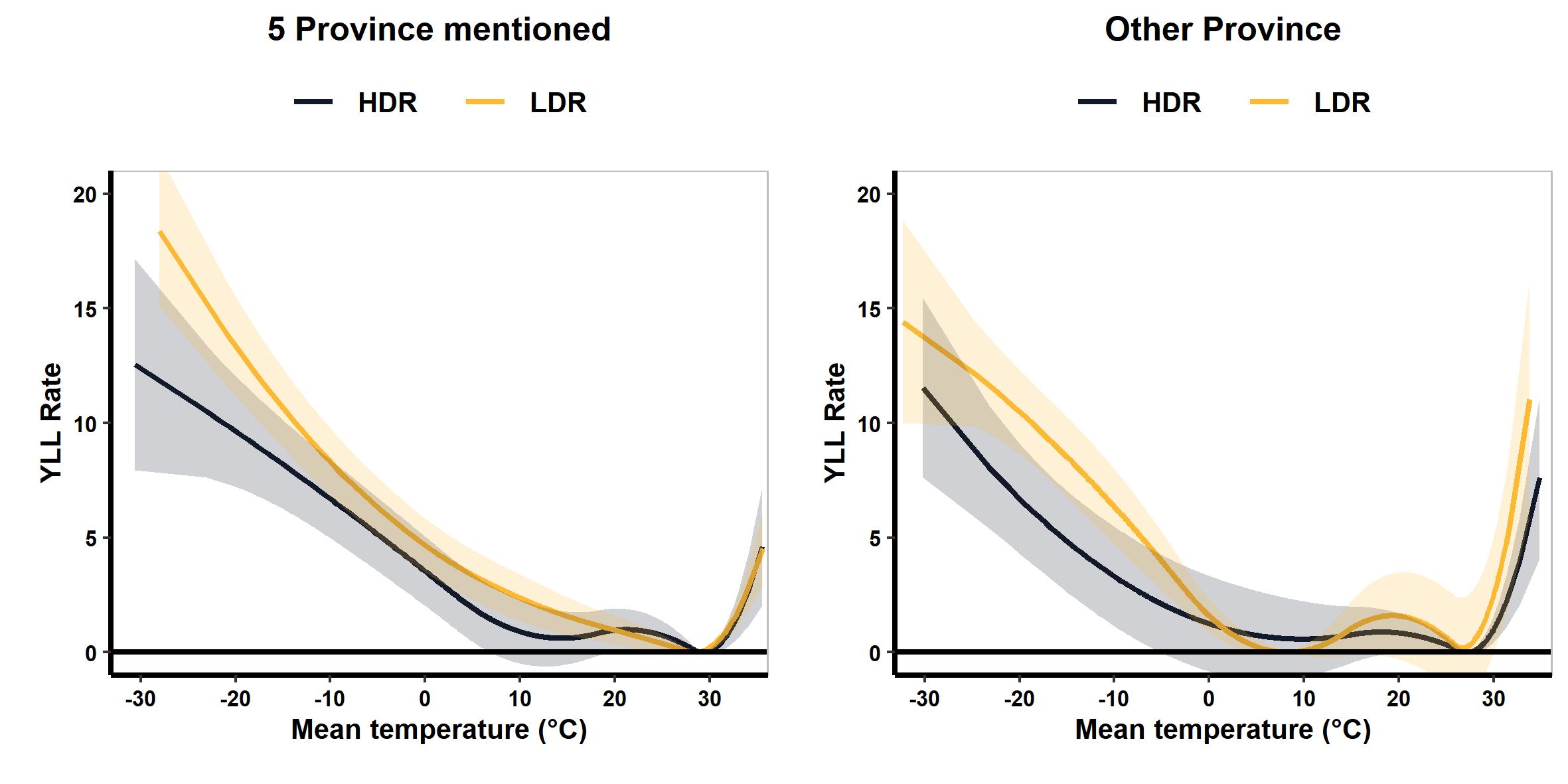


## Figure S5 Pooled temperature–YLL rate associations using different data source

YLL rate: years of life lost per 10^5^ population; HDR: high-development region; LDR: low-development region; *df*: degrees of freedom

**
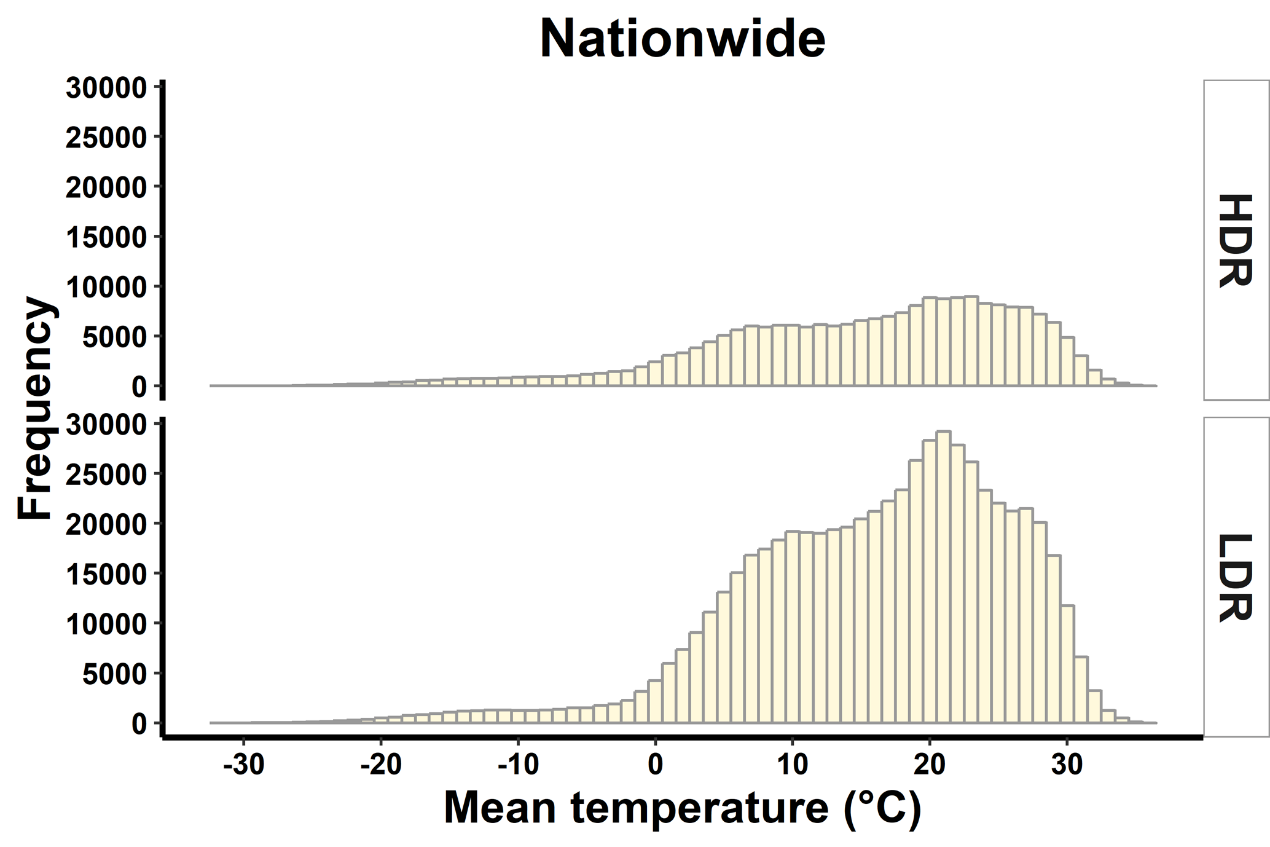
**

## Figure S6 Daily mean temperature distributions in China

HDR: high development region; LDR: low development region.

# Supplementary table

## Table S1 High development location and low development location

| **Development Level** | **Province** | **Locations** |
| --- | --- | --- |
| **High development** | Anhui | Daguan Anqing; |
|  | Beijing | Dongcheng |
|  | Fujian | Meilie Sanming; |
|  | Guangdong | Sanshui Foshan; Haizhu Guangzhou; Liwan Guangzhou; Yuexiu Guanghou; Huiyang Huizhou; Jianghai Jiangmen; Pengjiang Jiangmen; Qingcheng Qingyuan; Longhu Shantou; Wujiang Shaoguan; Zhenjiang Shaoguan; Duanzhou Zaoqing; |
|  | Guangxi | Xiufeng Guilin; |
|  | Henan | Jili Luoyang; Zhongyuan Zhengzhou |
|  | Heilongjiang | Nangang Haerbin |
|  | Hubei | Huangshigang Huangshi; Jiang’an Wuhan; Wujiagang Yichang |
|  | Hunan | Changsha Changsha; Furong Changsha; Kaifu Changsha; Yuhua Changsha; Yuelu Changsha; Louxing Loudi; Tianxin Changsha; Wuling Changde; Zhuhui Hengyang; Yuhu Xiangtan; |
|  | Jilin | Changyi Jilin; Chuangying Jilin; Fengman Jilin; Longshan Liaoyuan; Tiedong Siping; Tiexi Siping; Dongchang Tonghua; Chaoyang Changchun; Erdaoqu Changchun; Kuancheng Changchun; Nanguan Changchun; |
|  | Jiangsu | Pukou Nanjing; Wuzhong Suzhou; Yunlong Xuzhou; Zhangjiagang Suzhou; |
|  | Jiangxi | Donghu Nanchang; |
|  | Liaoning | Shahekou Dalian; Shenbei Shenyang |
|  | Nei Monggol | Huimin Huhehaote |
|  | Ningxia | Xingqing Yinchuan |
|  | Qinghai | Chengzhong Xining |
|  | Tianjin | Hongqiao |
|  | Shandong | Penglai Yantai; Shibei Qingdao; Zhifu Yantai; |
|  | Shanxi | Xinghualing Taiyuan; S |
|  | Shannxi | Wangyi Tongchuan |
|  | Shanghai | Luwan; Songjiang |
|  | Sichuan | Qingyang Chengdu; |
|  | Xinjiang | Tianshan Wulumuqi; Xinhe Akesu; |
|  | Yunnan | Guandu Kuning; Panlong Kunming; Wuhua Kunning Xishan Kuming |
|  | Zhejiang | Binjiang Hangzhou; Gongshu Hangzhou; Jianggan Hangzhou; Shangcheng Hangzhou; Xihu Hangzhou; Xiacheng Hangzhou; Xiaoshan Hangzhou; Yuhang Hangzhou; Nanhu Jiaxing; Wucheng Jinhua; Yiwu Jinhua; Beilun Ningbo; Haishu Ningbo; Jiangbei Ningbo; Jinzhou Ningbo; Zhenhai Ningbo; Shaoxing Shaoxing; Longwan Wenzhou; Lucheng Wenzhou; Ouhai Wenzhou; Dinghai Zhoushan |
| **Low development locations** | Anhui | Jingxian Xuancheng; |
|  | Chongqing | Wanzhou |
|  | Fujian | Huian Quanzhou |
|  | Gansu | Dunhuang; Ganzhou Zhangye; |
|  | Guangdong | Conghua Guangzhou; Heping Heyuan; Boluo Huizhou; Longmen Huizhou; Enping Jiangmen; Heshan Jiangmen; Kaiping Jiangmen; Taishan Jiangmen; Xinhui Jiangmen; Gaozhou Maoming; Wuhua Meizhou; Fogang Qingyuan; Liannan Qingyuan; Lianzhou Qingyuan; Qingxin Qingyuan; Yangshan Qingyuan; Yingde Qingyuan; Shanwei; Lechang Shaoguan; Nanxiong Shaoguan; Shixing Shaoguan; Wenyuan Shaoyuan; Yangdong Yangjiang; Luoding Yunfu; Yuncheng Yunfu; Wuchuan Zhanjiang; Sihui Zhaoqing; Doumen Zhuhai |
|  | Guangxi | Bingyang Nanning; Hepu Beihai; Lingyun Baise; |
|  | Guizhou | Meitan Zunyi; Honghuagang Zhunyi |
|  | Hebei | Qianxi Tangshan; Xuanhua Zhangjiakou |
|  | Henan | Hua Anyang; Tanghe Nanyang; Shihe Xinyang; |
|  | Heilongjiang | Datong Daqing; Huachuan Jiamusi; Yi’an Qiqihaer; |
|  | Hubei | Gucheng Xiangyang; |
|  | Hunan | Anxiang Changde; Dingcheng Changde; Hanshou Changde; Linli Changde; Liuyang Changsha; Shimen Changde; Taoyuan Changde; Ningxiang Changsha; Wangcheng Changsha; Anren Chenzhou; Linwu Chenzhou; Suxian Chenzhou; Yizhang Chenzhou; Yingxing Chenzhou; Changning Hengyang; Hengdong Hengyang; Hengnan Hengyang; Hengyang Heyang; Hengshan Hengyang; Leiyang Hengyang; Qidong Hengyang; Hongjiang Huaihua; Huitong Huaihua; Mayang Huaihua; Xupu Huaihua; Lianyuan Loudi; Louxing Loudi; Shuangfeng Loudi; Xinhua Loudi; Fenghuang Tujiazu; Luxi Tujiazu; Longhui Shaoyang; Dongkou Shaoyang; Shaodong Shaoyang; Shaoyang Shao Yang; Xinshao Shaoyang; Wugang Shaoyang; Xiangtan Xiangtan; Xiangxiang Xiangtan; Heshan Yiyang; Nanxian Yiyang; Taojiang Yiyang; Ziyang Yiyang; Daoxian Yongzhou; Jianghua Yongzhou; Dongan Yongzhou; Lanshan Yongzhou; Lengshuitan Yongzhou; Lingling Yongzhou; Qiyang Yongzhou; Yuanjiang Yuanjiang; Huarong Yueyang; Linxiang Yueyang; Pingjiang Yueyang; Xiangyin Yueyang; Yueyang Yueyang; Yueyanglou Yueyang; Cili Zhangjiajie; Sangzhi Zhangjiajie; Yongding Zhangjiajie; Chaling Zhuzhou; Liling Zhuzhou; Lusong Zhuzhou; Youxian Zhuzhou; Zhuzhou Zhuzhou |
|  | Jilin | Fusong Baishan; Huadian Jilin; Jiaohe Jilin; Panshi Jilin; Shulan Jilin; Yongji Jilin; Dongfeng Liaoyuan; Xi’an Liaoyuan; Ji’an Tonghua; Meihekou Tonthua; Tonghua Tonghua; Antu Yanbian; Dunhua Yanbian; Helong Yanbian; Huichun Yanbian; Longjing Yanbian; Wangqing Yanbian; Yanji Yanbian; Dehui Changchun; Shuangyang Changchun; |
|  | Jiangsu | Jinhu Huaian; Xiangshui Yancheng |
|  | Jiangxi | Wuning Jiujiang; |
|  | Liaoning | Fengcheng Dandong; Mengguzu Fuxin |
|  | Nei Monggol | Balin Chifeng; Linhe Bayanzhuoer; |
|  | Ningxia | Zhongwei Zhongwei |
|  | Qinghai | Menyuan Haibei |
|  | Shandong | Gaomi Weifang; Xuecheng Zaozhuang; |
|  | Shanxi | Jiangxian Yuncheng |
|  | Shannxi | Hanyin Ankang; Meixian Baoji; |
|  | Sichuan | Kangding Ganzi; Renhe Panzhihua; Pengzhou Chengdu; Xichong Nanchong; Zizhong Neijiang; |
|  | Xizang | Chengguan Lasha; Milin Linzhi; Mozugongka Lasha; |
|  | Yunnan | Longling Baoshan; Longyang Baoshan; Tengchong Baoshan; Chuxiong Chuxioing; Dayao Chuxiong; Lufeng Chuxiong; Wuding Chuxiong; Binchuan Dali; Dali Dali; Eryuan Dali; Midu Dali; Weishan Dali; Xiangyun Dali; Mangshi Dehong; Yingjiang Dehong; Gejiu Honghe; Honghe Honghe; Jianshui Honghe; Jinping Honghe; Kaiyuan Honghe; Luxi Honghe; Mengzi Honghe; Shiping Honghe; Yuanyang Honghe; Anning Kuning; Dongchuan Kunning; Jining Kuming; Luquan Kuming; Shilin Kuning; Songming Kunming; Xundian Kunning; Yiliang Kuming; Fengqing Linchang; Linxiang Lingchang; Jingdong Puer; Fuyuan Qujing; Huize Qujing; Huize Qujing; Luliang Qujing; Qilin Qujing; Xuanwei Qujing; Zhanyi Qujing Guangnan Wenshan; Wenshan Wenshan; Jinghong Xishuangbanna; Mengla Xishuangbanna; Jiangchuan Yuxi; Tonghai Yuxi; Xinping Yuxi; Zhaoyang Zhaotong; Zhenxiong Zhaotong; |
|  | Zhejiang | Chunan Hangzhou; Fuyang Hangzhou; Jiande Hangzhou; Lin’an Hangzhou; Tonglu Hangzhou; Anji Huzhou; Deqing Huzhou; Nanxun Huzhou; Wuxing Huzhou; Changxing Huzhou; Haining Jiaxing; Haiyan Jiaxing; Jiashan Jiaxing; Pinghu Jiaxing; Tongxiang Jiaxing; Tongxiang Jiaxing; Xiuzhou Jiaxing; Dongyang Jinhua; Jindong Jinhua; Lanxi Jinhua; Pan’an Jinhua; Pujiang Jinhua; Wuyi Jinhua; Yongkang Jinhua; Jinyun Lishui; Jingning Lishui; Liandu Lishui; Longquan Lishui; Qingtian Lishui; Qingyuan Lishui; Qingyuan Lishui; Qingyuan Lishui; Songyang Lishui; Suichang Lishui; Yunhe Lishui; Cixi Ningbo; Fenghua Ningbo; Ninghai Ningbo; Xiangshan Ningbo; Yuyao Ningbo; Changshan Quzhou; Jiangshan Quzhou; Kaihua Quzhou; Kecheng Quzhou; Longyou Quzhou; Qujiang Quzhou; Shangyu Shaoxing; Shengzhou Shaoxing; Xinchang Shaoxing; Yuecheng Shaoxing; Zhuji Shi; Huangyan Taizhou; Jiaojiang Taizhou; Linhai Taizhou; Luqiao Taizhou; Sanmen Taizhou; Tiantai Taizhou; Wenling Taizhou; Xianju Taizhou; Yuhuan Taizhou; Cangnan Wenzhou; Dongtou Wenzhou; Leqing Wenzhou; Pingyang Wenzhou; Ruian Wenzhou; Taishun Wenzhou; Wencheng Wenzhou; Yongjia Wenzhou; Daishan Zhoushan; Putuo Zhoushan; Shengsi Zhoushan |

## Table S2 Life table for provinces in China by age and sex

| **Province** |  | **0** | **1-4** | **5-9** | **10-14** | **15-19** | **20-24** | **25-29** | **30-34** | **35-39** | **40-44** | **45-49** | **50-54** | **55-59** | **60-64** | **65-69** | **70-74** | **75-79** | **80-84** | **85-89** | **90-94** | **95-99** | **100+** |
| --- | --- | --- | --- | --- | --- | --- | --- | --- | --- | --- | --- | --- | --- | --- | --- | --- | --- | --- | --- | --- | --- | --- | --- |
| **Nationwide** | **Both sexes** | 78.16 | 77.50 | 73.70 | 68.80 | 63.90 | 59.03 | 54.17 | 49.32 | 44.52 | 39.77 | 35.09 | 30.50 | 26.11 | 21.83 | 17.82 | 14.18 | 11.09 | 8.48 | 6.51 | 5.03 | 4.04 | 2.18 |
|  | **Male** | 75.86 | 75.19 | 71.39 | 66.51 | 61.63 | 56.79 | 51.98 | 47.19 | 42.44 | 37.76 | 33.18 | 28.70 | 24.44 | 20.33 | 16.49 | 13.04 | 10.16 | 7.77 | 5.99 | 4.72 | 3.99 | 1.90 |
|  | **Female** | 80.68 | 80.04 | 76.22 | 71.31 | 66.39 | 61.47 | 56.56 | 51.66 | 46.78 | 41.94 | 37.16 | 32.44 | 27.87 | 23.41 | 19.19 | 15.29 | 11.93 | 9.04 | 6.85 | 5.18 | 4.06 | 2.29 |
| **Beijing** | **Both sexes** | 81.96 | 81.07 | 77.14 | 72.17 | 67.23 | 62.28 | 57.33 | 52.37 | 47.43 | 42.53 | 37.70 | 32.97 | 28.36 | 23.85 | 19.53 | 15.51 | 11.95 | 8.91 | 6.49 | 4.63 | 3.45 | 2.18 |
|  | **Male** | 80.10 | 79.21 | 75.29 | 70.33 | 65.39 | 60.46 | 55.53 | 50.59 | 45.66 | 40.79 | 36.00 | 31.34 | 26.85 | 22.47 | 18.30 | 14.48 | 11.14 | 8.31 | 6.00 | 4.22 | 3.20 | 1.57 |
|  | **Female** | 83.92 | 83.03 | 79.09 | 74.12 | 69.16 | 64.19 | 59.21 | 54.25 | 49.28 | 44.36 | 39.47 | 34.64 | 29.89 | 25.24 | 20.75 | 16.52 | 12.75 | 9.47 | 6.90 | 4.94 | 3.62 | 2.54 |
| **Tianjin** | **Both sexes** | 81.64 | 80.76 | 76.82 | 71.86 | 66.93 | 62.01 | 57.06 | 52.13 | 47.20 | 42.37 | 37.59 | 32.87 | 28.26 | 23.74 | 19.46 | 15.55 | 12.21 | 9.43 | 7.28 | 5.70 | 4.67 | 2.43 |
|  | **Male** | 80.31 | 79.45 | 75.51 | 70.56 | 65.65 | 60.75 | 55.82 | 50.90 | 45.98 | 41.17 | 36.43 | 31.77 | 27.26 | 22.84 | 18.68 | 14.89 | 11.69 | 9.09 | 7.17 | 5.87 | 5.01 | 2.76 |
|  | **Female** | 83.06 | 82.16 | 78.20 | 73.25 | 68.29 | 63.34 | 58.38 | 53.42 | 48.48 | 43.62 | 38.78 | 33.99 | 29.28 | 24.63 | 20.22 | 16.18 | 12.70 | 9.74 | 7.38 | 5.60 | 4.47 | 2.27 |
| **Hebei** | **Both sexes** | 76.59 | 75.80 | 71.92 | 66.99 | 62.07 | 57.22 | 52.36 | 47.52 | 42.69 | 37.93 | 33.24 | 28.64 | 24.23 | 19.98 | 16.02 | 12.44 | 9.57 | 7.23 | 5.55 | 4.17 | 3.17 | 1.44 |
|  | **Male** | 74.35 | 73.56 | 69.68 | 64.77 | 59.87 | 55.07 | 50.28 | 45.50 | 40.73 | 36.05 | 31.44 | 26.96 | 22.68 | 18.59 | 14.80 | 11.41 | 8.72 | 6.54 | 5.01 | 3.79 | 3.02 | 0.85 |
|  | **Female** | 79.05 | 78.26 | 74.36 | 69.41 | 64.47 | 59.56 | 54.63 | 49.72 | 44.82 | 39.98 | 35.18 | 30.46 | 25.89 | 21.45 | 17.28 | 13.46 | 10.33 | 7.77 | 5.91 | 4.37 | 3.24 | 1.71 |
| **Shanxi** | **Both sexes** | 77.05 | 76.39 | 72.52 | 67.59 | 62.67 | 57.78 | 52.91 | 48.05 | 43.23 | 38.44 | 33.73 | 29.11 | 24.66 | 20.40 | 16.46 | 12.93 | 10.03 | 7.61 | 5.92 | 4.57 | 3.52 | 1.22 |
|  | **Male** | 75.01 | 74.33 | 70.46 | 65.56 | 60.64 | 55.79 | 50.98 | 46.18 | 41.42 | 36.72 | 32.09 | 27.55 | 23.21 | 19.07 | 15.27 | 11.91 | 9.21 | 6.98 | 5.47 | 4.28 | 3.38 | 0.81 |
|  | **Female** | 79.36 | 78.73 | 74.85 | 69.91 | 64.97 | 60.03 | 55.09 | 50.17 | 45.27 | 40.40 | 35.60 | 30.87 | 26.29 | 21.88 | 17.76 | 13.99 | 10.83 | 8.14 | 6.26 | 4.75 | 3.60 | 1.43 |
| **Nei Monggol** | **Both sexes** | 78.48 | 77.82 | 73.95 | 69.02 | 64.08 | 59.19 | 54.33 | 49.49 | 44.67 | 39.91 | 35.21 | 30.63 | 26.22 | 21.96 | 17.97 | 14.39 | 11.39 | 8.87 | 7.01 | 5.60 | 4.74 | 2.02 |
|  | **Male** | 76.27 | 75.65 | 71.79 | 66.86 | 61.94 | 57.07 | 52.26 | 47.48 | 42.73 | 38.05 | 33.46 | 29.01 | 24.75 | 20.66 | 16.87 | 13.52 | 10.75 | 8.45 | 6.71 | 5.39 | 4.67 | 1.69 |
|  | **Female** | 81.04 | 80.34 | 76.45 | 71.52 | 66.57 | 61.64 | 56.72 | 51.81 | 46.91 | 42.04 | 37.22 | 32.48 | 27.87 | 23.41 | 19.20 | 15.36 | 12.10 | 9.32 | 7.29 | 5.78 | 4.79 | 2.29 |
| **Liaoning** | **Both sexes** | 78.42 | 77.64 | 73.75 | 68.82 | 63.90 | 59.01 | 54.13 | 49.27 | 44.43 | 39.67 | 35.01 | 30.47 | 26.09 | 21.81 | 17.76 | 14.07 | 10.88 | 8.20 | 6.10 | 4.49 | 3.47 | 2.13 |
|  | **Male** | 76.26 | 75.50 | 71.62 | 66.71 | 61.80 | 56.96 | 52.12 | 47.30 | 42.52 | 37.84 | 33.31 | 28.92 | 24.73 | 20.64 | 16.77 | 13.28 | 10.29 | 7.80 | 5.82 | 4.26 | 3.40 | 2.17 |
|  | **Female** | 80.76 | 79.95 | 76.04 | 71.10 | 66.16 | 61.23 | 56.30 | 51.38 | 46.48 | 41.62 | 36.82 | 32.10 | 27.51 | 23.02 | 18.76 | 14.85 | 11.44 | 8.57 | 6.35 | 4.67 | 3.52 | 2.11 |
| **Jilin** | **Both sexes** | 79.72 | 78.85 | 74.94 | 69.99 | 65.05 | 60.15 | 55.27 | 50.40 | 45.54 | 40.76 | 36.07 | 31.49 | 27.09 | 22.87 | 18.93 | 15.41 | 12.45 | 9.98 | 8.08 | 6.55 | 5.26 | 2.81 |
|  | **Male** | 77.79 | 76.93 | 73.02 | 68.08 | 63.15 | 58.28 | 53.43 | 48.60 | 43.80 | 39.09 | 34.48 | 30.04 | 25.78 | 21.72 | 17.95 | 14.61 | 11.82 | 9.54 | 7.80 | 6.36 | 5.17 | 2.70 |
|  | **Female** | 81.80 | 80.92 | 77.01 | 72.06 | 67.10 | 62.17 | 57.25 | 52.33 | 47.42 | 42.56 | 37.76 | 33.04 | 28.47 | 24.06 | 19.94 | 16.24 | 13.09 | 10.41 | 8.35 | 6.71 | 5.34 | 2.90 |
| **Heilongjiang** | **Both sexes** | 79.29 | 78.41 | 74.50 | 69.56 | 64.62 | 59.72 | 54.83 | 49.96 | 45.11 | 40.33 | 35.65 | 31.09 | 26.71 | 22.49 | 18.61 | 15.22 | 12.41 | 10.07 | 8.30 | 6.80 | 5.25 | 2.18 |
|  | **Male** | 76.96 | 76.10 | 72.19 | 67.25 | 62.32 | 57.45 | 52.60 | 47.77 | 42.97 | 38.27 | 33.69 | 29.26 | 25.04 | 20.98 | 17.28 | 14.10 | 11.55 | 9.44 | 7.85 | 6.66 | 5.40 | 2.49 |
|  | **Female** | 81.91 | 81.02 | 77.10 | 72.16 | 67.20 | 62.26 | 57.34 | 52.42 | 47.51 | 42.64 | 37.84 | 33.12 | 28.56 | 24.16 | 20.09 | 16.46 | 13.37 | 10.75 | 8.74 | 6.95 | 5.17 | 2.01 |
| **Shanghai** | **Both sexes** | 82.36 | 81.62 | 77.74 | 72.80 | 67.86 | 62.92 | 57.97 | 53.02 | 48.08 | 43.17 | 38.29 | 33.53 | 28.92 | 24.38 | 19.98 | 15.82 | 12.11 | 8.89 | 6.35 | 4.47 | 3.18 | 1.92 |
|  | **Male** | 80.39 | 79.69 | 75.83 | 70.90 | 65.96 | 61.03 | 56.10 | 51.16 | 46.23 | 41.33 | 36.48 | 31.77 | 27.25 | 22.82 | 18.56 | 14.56 | 11.09 | 8.11 | 5.90 | 4.24 | 3.22 | 1.84 |
|  | **Female** | 84.36 | 83.58 | 79.69 | 74.73 | 69.79 | 64.84 | 59.87 | 54.90 | 49.95 | 45.02 | 40.11 | 35.29 | 30.57 | 25.88 | 21.30 | 16.93 | 12.94 | 9.47 | 6.63 | 4.60 | 3.16 | 1.94 |
| **Jiangsu** | **Both sexes** | 79.37 | 78.55 | 74.68 | 69.75 | 64.84 | 59.92 | 55.01 | 50.11 | 45.24 | 40.43 | 35.70 | 31.04 | 26.58 | 22.23 | 18.08 | 14.26 | 10.93 | 8.08 | 5.92 | 4.36 | 3.47 | 1.78 |
|  | **Male** | 77.19 | 76.37 | 72.52 | 67.60 | 62.70 | 57.81 | 52.92 | 48.06 | 43.23 | 38.47 | 33.81 | 29.23 | 24.90 | 20.70 | 16.72 | 13.09 | 9.95 | 7.33 | 5.39 | 4.07 | 3.50 | 1.56 |
|  | **Female** | 81.66 | 80.83 | 76.94 | 71.99 | 67.06 | 62.12 | 57.18 | 52.25 | 47.33 | 42.47 | 37.66 | 32.90 | 28.29 | 23.75 | 19.39 | 15.32 | 11.73 | 8.61 | 6.22 | 4.49 | 3.45 | 1.85 |
| **Zhejiang** | **Both sexes** | 79.61 | 78.94 | 75.15 | 70.25 | 65.32 | 60.42 | 55.52 | 50.63 | 45.76 | 40.93 | 36.16 | 31.48 | 26.99 | 22.60 | 18.38 | 14.44 | 10.99 | 7.96 | 5.67 | 4.00 | 3.04 | 1.87 |
|  | **Male** | 77.55 | 76.88 | 73.11 | 68.22 | 63.31 | 58.44 | 53.58 | 48.71 | 43.89 | 39.10 | 34.41 | 29.81 | 25.46 | 21.23 | 17.18 | 13.46 | 10.24 | 7.42 | 5.31 | 3.81 | 3.01 | 1.64 |
|  | **Female** | 81.92 | 81.25 | 77.44 | 72.53 | 67.58 | 62.64 | 57.71 | 52.77 | 47.86 | 42.97 | 38.13 | 33.35 | 28.69 | 24.11 | 19.67 | 15.46 | 11.71 | 8.42 | 5.92 | 4.10 | 3.05 | 1.96 |
| **Anhui** | **Both sexes** | 78.53 | 77.91 | 74.06 | 69.15 | 64.24 | 59.36 | 54.50 | 49.66 | 44.87 | 40.12 | 35.44 | 30.81 | 26.40 | 22.08 | 18.00 | 14.27 | 11.18 | 8.58 | 6.86 | 5.48 | 4.68 | 3.30 |
|  | **Male** | 76.13 | 75.49 | 71.66 | 66.76 | 61.87 | 57.02 | 52.22 | 47.44 | 42.71 | 38.04 | 33.44 | 28.91 | 24.61 | 20.42 | 16.52 | 12.98 | 10.10 | 7.72 | 6.15 | 4.89 | 4.21 | 2.38 |
|  | **Female** | 81.18 | 80.58 | 76.70 | 71.77 | 66.83 | 61.91 | 57.00 | 52.10 | 47.24 | 42.41 | 37.63 | 32.90 | 28.34 | 23.86 | 19.55 | 15.53 | 12.11 | 9.20 | 7.26 | 5.74 | 4.84 | 3.59 |
| **Fujian** | **Both sexes** | 78.82 | 78.11 | 74.23 | 69.31 | 64.40 | 59.52 | 54.64 | 49.78 | 44.95 | 40.17 | 35.46 | 30.83 | 26.43 | 22.12 | 18.04 | 14.30 | 11.06 | 8.29 | 6.18 | 4.51 | 3.54 | 2.01 |
|  | **Male** | 76.43 | 75.69 | 71.82 | 66.90 | 62.00 | 57.17 | 52.33 | 47.52 | 42.73 | 38.01 | 33.38 | 28.86 | 24.62 | 20.52 | 16.65 | 13.12 | 10.14 | 7.66 | 5.80 | 4.31 | 3.57 | 2.05 |
|  | **Female** | 81.51 | 80.82 | 76.94 | 72.00 | 67.07 | 62.15 | 57.22 | 52.32 | 47.43 | 42.58 | 37.77 | 33.01 | 28.39 | 23.85 | 19.49 | 15.43 | 11.86 | 8.75 | 6.40 | 4.60 | 3.53 | 2.00 |
| **Jiangxi** | **Both sexes** | 77.34 | 76.68 | 72.91 | 68.04 | 63.15 | 58.27 | 53.39 | 48.55 | 43.75 | 39.00 | 34.35 | 29.77 | 25.36 | 21.07 | 17.07 | 13.49 | 10.55 | 8.08 | 6.33 | 4.96 | 3.97 | 1.62 |
|  | **Male** | 74.81 | 74.10 | 70.34 | 65.49 | 60.62 | 55.76 | 50.92 | 46.13 | 41.41 | 36.73 | 32.18 | 27.71 | 23.44 | 19.31 | 15.50 | 12.12 | 9.42 | 7.18 | 5.64 | 4.49 | 3.86 | 1.50 |
|  | **Female** | 80.20 | 79.58 | 75.82 | 70.91 | 65.99 | 61.07 | 56.16 | 51.26 | 46.39 | 41.56 | 36.79 | 32.07 | 27.48 | 23.00 | 18.75 | 14.88 | 11.60 | 8.81 | 6.79 | 5.21 | 4.02 | 1.67 |
| **Shandong** | **Both sexes** | 78.45 | 77.61 | 73.70 | 68.77 | 63.84 | 58.95 | 54.07 | 49.21 | 44.37 | 39.59 | 34.88 | 30.26 | 25.85 | 21.56 | 17.51 | 13.81 | 10.72 | 8.10 | 6.06 | 4.57 | 3.55 | 1.97 |
|  | **Male** | 76.06 | 75.22 | 71.31 | 66.38 | 61.48 | 56.61 | 51.78 | 46.97 | 42.18 | 37.46 | 32.83 | 28.32 | 24.06 | 19.92 | 16.06 | 12.56 | 9.66 | 7.25 | 5.39 | 4.15 | 3.43 | 1.91 |
|  | **Female** | 80.97 | 80.14 | 76.22 | 71.28 | 66.33 | 61.40 | 56.47 | 51.56 | 46.67 | 41.81 | 37.01 | 32.27 | 27.68 | 23.19 | 18.91 | 14.95 | 11.59 | 8.70 | 6.47 | 4.77 | 3.60 | 1.99 |
| **Henan** | **Both sexes** | 78.22 | 77.31 | 73.41 | 68.48 | 63.56 | 58.65 | 53.77 | 48.92 | 44.11 | 39.36 | 34.69 | 30.11 | 25.76 | 21.54 | 17.64 | 14.12 | 11.32 | 8.95 | 7.30 | 5.91 | 4.79 | 2.69 |
|  | **Male** | 75.45 | 74.54 | 70.65 | 65.73 | 60.83 | 55.96 | 51.13 | 46.34 | 41.60 | 36.93 | 32.36 | 27.90 | 23.68 | 19.60 | 15.84 | 12.50 | 9.86 | 7.69 | 6.19 | 5.10 | 4.42 | 2.59 |
|  | **Female** | 81.12 | 80.21 | 76.31 | 71.36 | 66.40 | 61.46 | 56.53 | 51.61 | 46.73 | 41.88 | 37.10 | 32.39 | 27.89 | 23.50 | 19.38 | 15.60 | 12.53 | 9.84 | 7.94 | 6.27 | 4.92 | 2.72 |
| **Hubei** | **Both sexes** | 78.66 | 77.94 | 74.11 | 69.20 | 64.30 | 59.40 | 54.52 | 49.67 | 44.83 | 40.07 | 35.36 | 30.74 | 26.31 | 21.99 | 17.93 | 14.25 | 11.20 | 8.63 | 6.81 | 5.51 | 4.51 | 1.94 |
|  | **Male** | 76.57 | 75.84 | 72.02 | 67.12 | 62.23 | 57.36 | 52.53 | 47.72 | 42.93 | 38.23 | 33.60 | 29.06 | 24.75 | 20.56 | 16.67 | 13.18 | 10.37 | 8.07 | 6.45 | 5.46 | 4.74 | 1.54 |
|  | **Female** | 80.95 | 80.25 | 76.40 | 71.47 | 66.54 | 61.61 | 56.69 | 51.78 | 46.89 | 42.06 | 37.27 | 32.54 | 27.97 | 23.49 | 19.22 | 15.28 | 11.92 | 9.04 | 7.03 | 5.52 | 4.38 | 2.11 |
| **Hunan** | **Both sexes** | 78.60 | 77.81 | 74.04 | 69.15 | 64.26 | 59.37 | 54.51 | 49.66 | 44.85 | 40.11 | 35.44 | 30.85 | 26.51 | 22.25 | 18.23 | 14.59 | 11.47 | 8.87 | 6.85 | 5.32 | 4.24 | 2.07 |
|  | **Male** | 76.43 | 75.62 | 71.87 | 67.01 | 62.13 | 57.28 | 52.45 | 47.66 | 42.91 | 38.23 | 33.65 | 29.15 | 24.93 | 20.81 | 16.93 | 13.45 | 10.50 | 8.10 | 6.20 | 4.89 | 4.00 | 1.40 |
|  | **Female** | 81.04 | 80.25 | 76.46 | 71.55 | 66.62 | 61.70 | 56.79 | 51.89 | 47.02 | 42.20 | 37.44 | 32.74 | 28.24 | 23.81 | 19.59 | 15.73 | 12.37 | 9.51 | 7.30 | 5.58 | 4.37 | 2.45 |
| **Guangdong** | **Both sexes** | 79.29 | 78.53 | 74.67 | 69.75 | 64.83 | 59.92 | 55.01 | 50.11 | 45.23 | 40.41 | 35.66 | 31.02 | 26.60 | 22.30 | 18.24 | 14.55 | 11.40 | 8.72 | 6.70 | 5.12 | 4.03 | 1.98 |
|  | **Male** | 76.83 | 76.05 | 72.19 | 67.28 | 62.37 | 57.49 | 52.61 | 47.73 | 42.89 | 38.11 | 33.43 | 28.87 | 24.59 | 20.46 | 16.59 | 13.14 | 10.25 | 7.90 | 6.19 | 4.95 | 4.17 | 1.74 |
|  | **Female** | 82.05 | 81.31 | 77.45 | 72.51 | 67.56 | 62.63 | 57.69 | 52.75 | 47.84 | 42.96 | 38.13 | 33.38 | 28.77 | 24.25 | 19.93 | 15.90 | 12.37 | 9.32 | 6.99 | 5.19 | 3.96 | 2.05 |
| **Guangxi** | **Both sexes** | 78.59 | 77.90 | 74.09 | 69.21 | 64.31 | 59.44 | 54.61 | 49.81 | 45.07 | 40.40 | 35.84 | 31.36 | 27.06 | 22.88 | 18.96 | 15.40 | 12.32 | 9.68 | 7.64 | 6.09 | 4.67 | 2.53 |
|  | **Male** | 75.19 | 74.48 | 70.68 | 65.82 | 60.92 | 56.10 | 51.34 | 46.61 | 41.97 | 37.43 | 33.01 | 28.68 | 24.58 | 20.61 | 16.92 | 13.63 | 10.80 | 8.44 | 6.61 | 5.35 | 4.20 | 1.91 |
|  | **Female** | 82.45 | 81.79 | 77.97 | 73.06 | 68.14 | 63.22 | 58.31 | 53.41 | 48.54 | 43.72 | 38.98 | 34.28 | 29.72 | 25.26 | 21.01 | 17.07 | 13.63 | 10.63 | 8.30 | 6.47 | 4.87 | 2.76 |
| **Hainan** | **Both sexes** | 81.57 | 81.08 | 77.30 | 72.40 | 67.51 | 62.65 | 57.79 | 52.97 | 48.15 | 43.39 | 38.71 | 34.08 | 29.59 | 25.25 | 21.18 | 17.47 | 14.24 | 11.31 | 9.08 | 7.48 | 6.31 | 5.67 |
|  | **Male** | 78.60 | 78.04 | 74.27 | 69.39 | 64.51 | 59.71 | 54.91 | 50.14 | 45.39 | 40.71 | 36.11 | 31.60 | 27.24 | 23.05 | 19.16 | 15.73 | 12.79 | 10.21 | 8.34 | 7.16 | 6.67 | 6.38 |
|  | **Female** | 84.86 | 84.48 | 80.67 | 75.74 | 70.83 | 65.90 | 60.98 | 56.09 | 51.19 | 46.33 | 41.52 | 36.73 | 32.06 | 27.49 | 23.17 | 19.07 | 15.47 | 12.11 | 9.53 | 7.66 | 6.23 | 5.50 |
| **Chongqing** | **Both sexes** | 78.45 | 77.75 | 73.98 | 69.13 | 64.25 | 59.38 | 54.53 | 49.71 | 44.97 | 40.29 | 35.72 | 31.21 | 26.94 | 22.72 | 18.69 | 14.98 | 11.73 | 9.04 | 6.90 | 5.21 | 4.08 | 2.00 |
|  | **Male** | 76.05 | 75.35 | 71.60 | 66.77 | 61.92 | 57.08 | 52.29 | 47.52 | 42.87 | 38.29 | 33.85 | 29.48 | 25.43 | 21.40 | 17.56 | 14.03 | 10.97 | 8.53 | 6.60 | 5.10 | 3.98 | 1.66 |
|  | **Female** | 81.14 | 80.43 | 76.63 | 71.76 | 66.83 | 61.93 | 57.02 | 52.15 | 47.32 | 42.53 | 37.81 | 33.13 | 28.61 | 24.15 | 19.89 | 15.94 | 12.44 | 9.47 | 7.10 | 5.26 | 4.12 | 2.14 |
| **Sichuan** | **Both sexes** | 77.45 | 76.74 | 73.03 | 68.19 | 63.34 | 58.49 | 53.69 | 48.92 | 44.21 | 39.55 | 34.97 | 30.45 | 26.22 | 22.05 | 18.12 | 14.50 | 11.37 | 8.80 | 6.77 | 5.28 | 4.23 | 2.27 |
|  | **Male** | 75.06 | 74.35 | 70.66 | 65.84 | 61.01 | 56.20 | 51.46 | 46.76 | 42.15 | 37.58 | 33.11 | 28.73 | 24.68 | 20.70 | 16.96 | 13.53 | 10.59 | 8.24 | 6.37 | 5.07 | 4.31 | 2.13 |
|  | **Female** | 80.12 | 79.41 | 75.68 | 70.81 | 65.92 | 61.03 | 56.17 | 51.32 | 46.52 | 41.75 | 37.03 | 32.36 | 27.91 | 23.51 | 19.34 | 15.48 | 12.11 | 9.26 | 7.06 | 5.40 | 4.18 | 2.32 |
| **Guizhou** | **Both sexes** | 74.83 | 75.05 | 71.44 | 66.65 | 61.81 | 57.07 | 52.42 | 47.79 | 43.22 | 38.69 | 34.23 | 29.79 | 25.56 | 21.40 | 17.49 | 13.93 | 10.90 | 8.41 | 6.47 | 5.20 | 4.45 | 2.60 |
|  | **Male** | 72.14 | 72.21 | 68.62 | 63.86 | 59.05 | 54.37 | 49.84 | 45.34 | 40.92 | 36.55 | 32.24 | 27.98 | 23.94 | 19.97 | 16.26 | 12.90 | 10.09 | 7.81 | 6.02 | 4.97 | 4.37 | 2.25 |
|  | **Female** | 77.83 | 78.23 | 74.58 | 69.76 | 64.89 | 60.07 | 55.29 | 50.51 | 45.77 | 41.04 | 36.39 | 31.75 | 27.28 | 22.89 | 18.73 | 14.93 | 11.64 | 8.90 | 6.78 | 5.33 | 4.49 | 2.77 |
| **Yunan** | **Both sexes** | 73.66 | 73.79 | 70.36 | 65.55 | 60.73 | 55.97 | 51.26 | 46.58 | 41.95 | 37.37 | 32.86 | 28.43 | 24.21 | 20.09 | 16.32 | 12.89 | 10.04 | 7.61 | 5.81 | 4.62 | 3.75 | 1.69 |
|  | **Male** | 71.16 | 71.19 | 67.76 | 62.99 | 58.21 | 53.51 | 48.88 | 44.30 | 39.78 | 35.32 | 30.95 | 26.67 | 22.61 | 18.65 | 15.06 | 11.84 | 9.19 | 6.97 | 5.34 | 4.38 | 3.68 | 1.34 |
|  | **Female** | 76.51 | 76.74 | 73.30 | 68.46 | 63.59 | 58.75 | 53.94 | 49.14 | 44.38 | 39.64 | 34.95 | 30.34 | 25.91 | 21.59 | 17.59 | 13.91 | 10.80 | 8.11 | 6.13 | 4.75 | 3.78 | 1.87 |
| **Xizang** | **Both sexes** | 72.47 | 72.48 | 69.25 | 64.56 | 59.84 | 55.11 | 50.46 | 45.81 | 41.21 | 36.68 | 32.22 | 27.98 | 23.93 | 20.22 | 17.07 | 14.25 | 12.10 | 10.17 | 8.83 | 7.73 | 6.40 | 3.17 |
|  | **Male** | 70.53 | 70.51 | 67.26 | 62.54 | 57.82 | 53.10 | 48.43 | 43.79 | 39.20 | 34.67 | 30.23 | 26.06 | 22.10 | 18.47 | 15.43 | 12.75 | 10.72 | 8.85 | 7.70 | 7.04 | 5.88 | 2.43 |
|  | **Female** | 74.44 | 74.47 | 71.27 | 66.61 | 61.88 | 57.14 | 52.51 | 47.84 | 43.23 | 38.69 | 34.20 | 29.86 | 25.69 | 21.85 | 18.55 | 15.56 | 13.24 | 11.23 | 9.75 | 8.41 | 7.14 | 4.20 |
| **Shaanxi** | **Both sexes** | 77.76 | 76.92 | 73.09 | 68.18 | 63.27 | 58.39 | 53.53 | 48.70 | 43.90 | 39.15 | 34.48 | 29.89 | 25.46 | 21.20 | 17.25 | 13.71 | 10.76 | 8.28 | 6.60 | 5.37 | 4.59 | 2.07 |
|  | **Male** | 75.95 | 75.12 | 71.30 | 66.39 | 61.49 | 56.65 | 51.85 | 47.08 | 42.35 | 37.69 | 33.09 | 28.59 | 24.24 | 20.09 | 16.26 | 12.85 | 10.07 | 7.77 | 6.21 | 5.12 | 4.61 | 2.02 |
|  | **Female** | 79.74 | 78.91 | 75.07 | 70.14 | 65.22 | 60.30 | 55.38 | 50.48 | 45.60 | 40.76 | 36.00 | 31.30 | 26.77 | 22.39 | 18.28 | 14.58 | 11.44 | 8.76 | 6.91 | 5.54 | 4.58 | 2.10 |
| **Gansu** | **Both sexes** | 76.35 | 75.99 | 72.28 | 67.41 | 62.54 | 57.68 | 52.88 | 48.08 | 43.33 | 38.61 | 33.94 | 29.34 | 24.95 | 20.71 | 16.83 | 13.38 | 10.52 | 8.20 | 6.62 | 5.58 | 4.99 | 2.30 |
|  | **Male** | 74.62 | 74.18 | 70.46 | 65.59 | 60.73 | 55.91 | 51.15 | 46.42 | 41.73 | 37.09 | 32.50 | 27.99 | 23.69 | 19.55 | 15.81 | 12.52 | 9.83 | 7.70 | 6.22 | 5.32 | 4.72 | 1.81 |
|  | **Female** | 78.27 | 78.00 | 74.31 | 69.43 | 64.54 | 59.65 | 54.79 | 49.94 | 45.12 | 40.31 | 35.55 | 30.85 | 26.34 | 21.98 | 17.94 | 14.29 | 11.22 | 8.69 | 6.95 | 5.76 | 5.16 | 2.60 |
| **Qinghai** | **Both sexes** | 73.31 | 73.21 | 70.03 | 65.54 | 60.90 | 56.28 | 51.75 | 47.22 | 42.65 | 38.09 | 33.57 | 29.08 | 24.87 | 20.82 | 17.17 | 13.84 | 11.11 | 8.89 | 7.28 | 6.45 | 5.97 | 5.25 |
|  | **Male** | 71.38 | 71.30 | 68.13 | 63.67 | 59.05 | 54.48 | 50.00 | 45.50 | 40.97 | 36.48 | 32.06 | 27.68 | 23.59 | 19.65 | 16.19 | 13.10 | 10.55 | 8.52 | 7.04 | 6.54 | 6.40 | 5.33 |
|  | **Female** | 75.44 | 75.32 | 72.13 | 67.60 | 62.93 | 58.27 | 53.67 | 49.11 | 44.50 | 39.85 | 35.22 | 30.58 | 26.20 | 22.02 | 18.15 | 14.58 | 11.64 | 9.22 | 7.46 | 6.38 | 5.70 | 5.20 |
| **Ningxia** | **Both sexes** | 74.79 | 74.62 | 70.99 | 66.15 | 61.32 | 56.53 | 51.80 | 47.08 | 42.37 | 37.67 | 33.01 | 28.45 | 24.06 | 19.79 | 15.83 | 12.24 | 9.24 | 6.84 | 5.28 | 4.34 | 3.99 | 1.58 |
|  | **Male** | 72.76 | 72.61 | 68.97 | 64.17 | 59.39 | 54.68 | 50.05 | 45.43 | 40.81 | 36.21 | 31.67 | 27.24 | 22.99 | 18.84 | 15.03 | 11.60 | 8.77 | 6.57 | 5.08 | 4.12 | 3.82 | 0.95 |
|  | **Female** | 77.07 | 76.88 | 73.25 | 68.37 | 63.49 | 58.61 | 53.77 | 48.93 | 44.12 | 39.32 | 34.52 | 29.80 | 25.26 | 20.85 | 16.72 | 12.96 | 9.76 | 7.14 | 5.48 | 4.55 | 4.12 | 2.07 |
| **Xinjiang** | **Both sexes** | 77.95 | 77.61 | 74.06 | 69.23 | 64.37 | 59.57 | 54.81 | 50.06 | 45.33 | 40.60 | 35.93 | 31.38 | 27.09 | 23.08 | 19.47 | 16.19 | 13.23 | 10.76 | 8.88 | 7.36 | 5.67 | 3.06 |
|  | **Male** | 76.01 | 75.70 | 72.19 | 67.40 | 62.55 | 57.78 | 53.08 | 48.40 | 43.74 | 39.09 | 34.50 | 30.05 | 25.81 | 21.90 | 18.44 | 15.36 | 12.45 | 10.06 | 8.26 | 6.80 | 5.28 | 2.56 |
|  | **Female** | 80.36 | 79.97 | 76.39 | 71.51 | 66.64 | 61.80 | 56.97 | 52.16 | 47.34 | 42.53 | 37.75 | 33.10 | 28.73 | 24.61 | 20.85 | 17.38 | 14.34 | 11.77 | 9.76 | 8.08 | 6.18 | 3.73 |

## Table S3 Attributable fraction of YLL for separated temperature components in HDRs and LDRs.

| **Subgroups** | **Extreme heat** | | **Moderate heat** | | **Moderate cold** | | **Extreme cold** | |
| --- | --- | --- | --- | --- | --- | --- | --- | --- |
|  | **HDRs** | **LDRs** | **HDRs** | **LDRs** | **HDRs** | **LDRs** | **HDRs** | **LDRs** |
| Nationwide | 0.3(0.2-0.3) | 0.3(0.2-0.3) | 0.1(0.1-0.2) | 1.6(1.1-2.1) | 7.7(7.2-8.1) | 8.3(7.5-9.1) | 1.0(1.0-1.1) | 1.1(1.0-1.2) |
| North China | 0.5(0.4-0.6) | 0.40.3-0.6) | 1.8(0.5-3.0) | 4.7(2.5-7.0) | 3.6(1.7-5.4) | 5.2(3.6-6.9) | 0.8(0.7-0.9) | 0.9(0.7-1.1) |
| South China | 0.2(0.2-0.3) | 0.3(0.2-0.3) | 0.1(0.0-0.1) | 1.5(1.0-2.0) | 8.8(8.3-9.4) | 8.8(7.9-9.8) | 1.1(1.0-1.2) | 1.1(1.1-1.2) |
| Age 0-64 | 0.2(0.1-0.2) | 0.2(0.1-0.2) | 0.3(-0.3-0.9) | 1.2(0.8-1.6) | 5.4(4.1-6.9) | 5.2(4.2-6.0) | 0.8(0.7-0.9) | 0.7(0.7-0.8) |
| Age ≥ 65 | 0.4(0.3-0.4) | 0.3(0.3-0.4) | 0.3(0.1-0.5) | 1.7(1.0-2.3) | 11.1(10.3-12.0) | 11.5(10.7-12.4) | 1.4(1.3-1.5) | 1.5(1.5-1.6) |
| Males | 0.2(0.2-0.2) | 0.2(0.2-0.3) | 0.2(0.0-0.5) | 0.9(0.4-1.3) | 8.8(7.5-10.2) | 8.2(7.5-9.0) | 1.0(0.9-1.1) | 1.0(0.9-1.1) |
| Females | 0.4(0.3-0.4) | 0.3(0.3-0.3) | 0.1(0.1-0.2) | 1.4(0.8-1.9) | 6.7(5.8-7.8) | 7.0(6.0-8.1) | 1.0(1.0-1.1) | 1.1(1.0-1.2) |
| Cardiovascular diseases | 0.4(0.4-0.5) | 0.3(0.3-0.4) | 0.9(0.5-1.4) | 0.8(0.1-1.5) | 7.7(6.7-8.8) | 10.7(9.9-11.3) | 1.4(1.3-1.5) | 1.4(1.4-1.5) |
| Respiratory diseases | 0.4(0.3-0.5) | 0.5(0.4-0.6) | 1.7(-0.7-3.6) | 4.3(1.9-6.7) | 9.7(7.9-11.4) | 6.2(5.3-7.0) | 1.6(1.4-1.8) | 1.5(1.4-1.6) |

HDRs: high-development regions; LDRs: low-development regions

## Table S4 Life loss per death for separated temperature components in HDRs and LDRs.

| **Subgroups** | **Extreme heat** | | **Moderate heat** | | **Moderate cold** | | **Extreme cold** | |
| --- | --- | --- | --- | --- | --- | --- | --- | --- |
|  | **HDRs** | **LDRs** | **HDRs** | **LDRs** | **HDRs** | **LDRs** | **HDRs** | **LDRs** |
| Nationwide | 0.04(0.04-0.05) | 0.05(0.04-0.05) | 0.02(0.01-0.03) | 0.24(0.13-0.35) | 1.11(1.02-1.91) | 1.45(1.29-1.60) | 0.15(0.14-0.16) | 0.17(0.16-0.18) |
| North China | 0.09(0.07-0.10) | 0.10(0.07-0.13) | 0.43(0.15-0.71) | 1.10(0.63-1.67) | 0.51(0.26-0.76) | 1.19(0.87-1.50) | 0.13(0.11-0.15) | 0.19(0.16-0.23) |
| South China | 0.03(0.03-0.04) | 0.05(0.04-0.05) | 0.01(0.01-0.02) | 0.24(0.11-0.36) | 1.23(1.09-1.35) | 1.48(1.30-1.64) | 0.15(0.15-0.17) | 0.17(0.16-0.18) |
| Age 0-64 | 0.06(0.04-0.08) | 0.07(0.05-0.08) | 0.33(-0.36-0.97) | 0.56(0.26-0.89) | 1.43(0.96-1.85) | 2.16(1.71-2.61) | 0.21(0.19-0.26) | 0.24(0.22-0.27) |
| Age ≥ 65 | 0.04(0.03-0.04) | 0.04(0.03-0.04) | 0.03(0.01-0.05) | 0.14(0.06-0.22) | 1.08(0.97-1.18) | 1.27(1.17-1.36) | 0.12(0.12-0.14) | 0.15(0.14-0.15) |
| Males | 0.03(0.03-0.04) | 0.22(0.04-0.41) | 0.06(0.00-0.11) | 0.22(0.04-0.41) | 1.33(1.11-1.51) | 1.47(1.32-1.61) | 0.15(0.15-0.18) | 0.16(0.15-0.17) |
| Females | 0.06(0.05-0.07) | 0.05(0.04-0.05) | 0.02(0.01-0.02) | 0.18(0.06-0.31) | 0.94(0.83-1.04) | 1.26(1.07-1.44) | 0.14(0.13-0.16) | 0.17(0.16-0.18) |
| Cardiovascular diseases | 0.06(0.05-0.07) | 0.05(0.04-0.05) | 0.14(0.06-0.21) | 0.25(0.04-0.45) | 1.04(0.85-1.21) | 1.43(1.32-1.53) | 0.17(0.17-0.20) | 0.18(0.17-0.20) |
| Respiratory diseases | 0.04(0.03-0.05) | 0.06(0.04-0.07) | 0.17(-0.06-0.40) | 0.59(0.21-0.95) | 0.95(0.74-1.15) | 0.72(0.61-0.82) | 0.15(0.14-0.18) | 0.16(0.15-0.17) |

HDRs: high-development regions; LDRs: low-development regions
